# Unraveling the Complexity of the Rhomboid Serine Protease 4 Family of *Babesia bovis* Using Bioinformatics and Experimental Studies

**DOI:** 10.3390/pathogens11030344

**Published:** 2022-03-12

**Authors:** Romina Gallenti, Hala E. Hussein, Heba F. Alzan, Carlos E. Suarez, Massaro Ueti, Sebastián Asurmendi, Daniel Benitez, Flabio R. Araujo, Peter Rolls, Kgomotso Sibeko-Matjila, Leonhard Schnittger, Mónica Florin-Christensen

**Affiliations:** 1 Instituto de Patobiología Veterinaria (IPVET), Centro de Investigaciones en Ciencias Veterinarias y Agronómicas, Instituto Nacional de Tecnología Agropecuaria—Consejo Nacional de Investigaciones Científicas y Técnicas (INTA-CONICET), Hurlingham 1686, Argentina; rjgallenti@gmail.com (R.G.); schnittger.leonhard@inta.gob.ar (L.S.); 2 Consejo Nacional de Investigaciones Científicas y Técnicas (CONICET), Buenos Aires C1033AAJ, Argentina; 3 Department of Veterinary Microbiology and Pathology, Washington State University, Pullman, WA 99164, USA; hala.elsayed@wsu.edu (H.E.H.); heba.alzan@wsu.edu (H.F.A.); suarez@wsu.edu (C.E.S.); massaro_ueti@wsu.edu (M.U.); 4 Department of Entomology, Faculty of Science, Cairo University, Giza 12613, Egypt; 5 Tick and Tick-Borne Disease Research Unit, National Research Center, Giza 12622, Egypt; 6 US Department of Agriculture, Animal Disease Research Unit, (USDA-ARS), Pullman, WA 99163, USA; 7 Instituto de Agrobiotecnología y Biología Molecular (IABiMo), Instituto Nacional de Tecnología Agropecuaria—Consejo Nacional de Investigaciones Científicas y Técnicas (INTA-CONICET), Hurlingham 1686, Argentina; asurmendi.sebastian@inta.gob.ar; 8 Estación Experimental Agropecuaria (EEA)-Mercedes, Instituto Nacional de Tecnología Agropecuaria (INTA), Mercedes 3470, Argentina; benitez.daniel@inta.gob.ar; 9 EMBRAPA Beef Cattle, Campo Grande 79106-550, Brazil; flabio.araujo@embrapa.br; 10 Department of Agriculture & Fisheries, Tick Fever Centre, Wacol, QLD 4076, Australia; peter.rolls@daf.qld.gov.au; 11 Department of Veterinary Tropical Diseases, Faculty of Veterinary Science, University of Pretoria, Onderstepoort 0110, South Africa; kgomotso.sibeko@up.ac.za

**Keywords:** tick-borne diseases, bovine babesiosis, rhomboid serine proteases, *Babesia bovis* gene expression, inter-strain polymorphism

## Abstract

*Babesia bovis*, a tick-transmitted apicomplexan protozoon, infects cattle in tropical and subtropical regions around the world. In the apicomplexans *Toxoplasma gondii* and *Plasmodium falciparum*, rhomboid serine protease 4 (ROM4) fulfills an essential role in host cell invasion. We thus investigated *B. bovis* ROM4 coding genes; their genomic organization; their expression in in vitro cultured asexual (AS) and sexual stages (SS); and strain polymorphisms. *B. bovis* contains five *rom4* paralogous genes in chromosome 2, which we have named *rom4.1*, *4.2*, *4.3*, *4.4* and *4.5*. There are moderate degrees of sequence identity between them, except for *rom4.3* and *4.4*, which are almost identical. RT-qPCR analysis showed that *rom4.1* and *rom4.3/4.4*, respectively, display 18-fold and 218-fold significantly higher (*p* < 0.01) levels of transcription in SS than in AS, suggesting a role in gametogenesis-related processes. In contrast, transcription of *rom4.4* and *4.5* differed non-significantly between the stages. ROM4 polymorphisms among geographic isolates were essentially restricted to the number of tandem repeats of a 29-amino acid sequence in ROM4.5. This sequence repeat is highly conserved and predicted as antigenic. *B. bovis* ROMs likely participate in relevant host–pathogen interactions and are possibly useful targets for the development of new control strategies against this pathogen.

## 1. Introduction

*Babesia bovis* is a highly pathogenic tick-transmitted apicomplexan protozoon causing bovine babesiosis, which affects the welfare and productivity of cattle in vast tropical and subtropical regions of the world [1,2,3].

The genus *Babesia* belongs to the order Piroplasmida, which is characterized by a dixenous life cycle, and alternating asexual reproduction in a vertebrate and sexual and asexual reproduction in an ixodid tick host. Similarly to other related apicomplexans, *B. bovis* is an obligate intracellular parasite, and thus the processes of invasion and egress from host cells are essential for its survival [4]. In the case of *B. bovis, Rhipicephalus microplus* is the most common vector worldwide [5]. In the vertebrate bovine host, *B. bovis*, pertaining to *Babesia* sensu stricto (Clade VI) [2,3], thrives and divides exclusively within erythrocytes by merogony. During a blood meal on an infected animal, parasitized erythrocytes are ingested by ticks, and in the tick’s gut, parasites are released and transformed into characteristic isogametic ray bodies. After gamete fusion, the resulting zygotes migrate across the gut epithelium into the hemolymph, undergo meiosis and mature into kinetes, which invade different tick tissues where they propagate by sporogony. Importantly, colonization and invasion of tick ovaries and oocytes allow vertical transmission of the parasite to the next tick generation (transovarial transmission). Upon kinete invasion of the epithelial cells of the tick salivary glands, additional sporogony results in the production of a large quantity of infective sporozoites. Sporozoites are injected with tick saliva into the bovine dermis upon a blood meal, reach the capillaries, invade erythrocytes and undergo asexual merogony, closing the cycle [3,4,6].

In naive adult cattle, the ensuing parasitemia is accompanied by fever and anemia. A particular feature of *B. bovis* infection is the accumulation of infected erythrocytes within capillaries, provoking ischemia, which leads to nervous signs and respiratory shock [5]. Infection of naïve adult animals is often fatal if untreated, and in some cases, fetal infection and abortion in pregnant females has been reported [5,7].

Many of the details of the mechanisms of pathogenicity and interactions of *B. bovis* with its bovine and tick hosts remain unknown, yet elucidation may potentially lead to the development of novel control strategies [8,9].

Proteases of several parasitic protozoa have been described as key players in host–pathogen interactions [10]. Among them, rhomboid serine proteases (ROMs) are particularly remarkable. They are characterized by several transmembrane domains (TMDs) folding through the membrane, where the active site lies; thus, notably, catalytic cleavage of substrates takes place within a hydrophobic environment. ROMs are present in all kingdoms of life, fulfilling a large variety of essential functions [11]. In *Plasmodium falciparum* and *Toxoplasma gondii,* nine ROM subfamilies altogether, designated ROM1, 2, 3, 4, 6, 7, 8, 9 and 10, have been described, although not all are present in both microorganisms. Of these, ROM4, a subfamily confined to Apicomplexan parasites, has attracted particular interest, since it has been shown to be involved in adhesin cleavage—that is, dismantling adhesive junctions formed between parasite and host cell membranes, allowing invasion of the host cell [12,13,14,15]. Importantly, in piroplasmids, only subfamilies ROM4, 6, 7, and 8 have been reported, and it has been hypothesized that ROM4 exerts a similar critical function in erythrocyte invasion [16].

We have previously shown that *B. bovis* contains five ROM4-coding paralog genes [16]. Functional studies are still pending on whether any of these ROM4 paralogs fulfills a similar role in invasion of host cells as their *P. falciparum* counterpart. Recent transcriptomic studies showed that two of the five *B. bovis rom4* genes display increased expression (highly significant) in the tick hemolymph compared to bovine blood stages. This finding suggests that ROM4′s functions in *B. bovis* extend beyond erythrocyte invasion, and may be associated with processes that occur in the tick milieu [17]. 

The present study was designed to characterize *rom4* gene paralogs of *B. bovis*, including their genomic positions, topologies and polymorphisms among geographic isolates. In addition, we analyzed whether a differential expression pattern, similar to the one found in bovine blood and tick stages, could also be observed in in vitro cultured and in in vitro induced sexual stages of *B. bovis*. 

## 2. Results

### 2.1. Genomic Location of rom4 Paralogs

**Figure 1:**
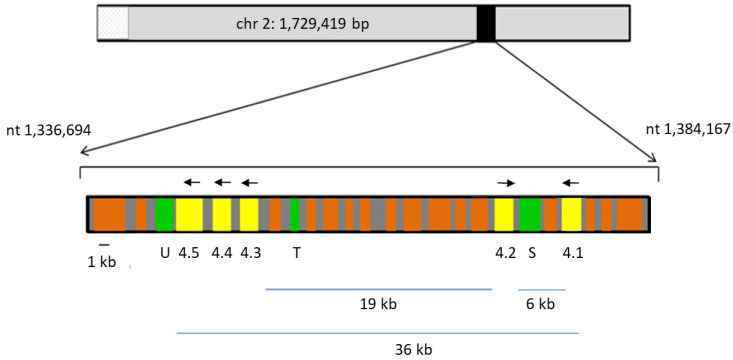
*Babesia bovis rom4* locus. A section of chromosome 2 (nt. 1,336,694 to 1,384,167) containing the paralogs in Table 1. (4.1, 4.2, 4.3, 4.4 and 4.5, yellow rectangles) is shown in scale. Genes coding for proteases or protease-related functions (U: ubiquitin fusion degradation protein; T: threonine protease of the 20S proteasome; S: subtilisin-like protease) are marked in green. *Orfs* not related to proteases are marked in orange and intergenic regions in grey. Arrows mark the orientation of the *rom4 orfs*.

The genome of *B. bovis* strain T2Bo contains five paralogous genes within a 36 kb-segment of chromosome (chr) 2, encoding four rhomboid serine proteases (ROMs), which we have designated ROM4.1, ROM4.2, ROM4.3, ROM4.4 and ROM4.5 (Figure 1; Table 1). *B. bovis rom4* genes have 3–7 introns (data not shown). *Rom4.3*, *4.4* and *4.5* genes are tandemly arranged head to tail, separated by 678 and 748 bp intergenic regions, respectively. A 6 kb segment, which contains two *orfs*, one of which corresponds to a subtilisin-like protease (XP_001610126), separates the oppositely-oriented *rom4.1* and *rom4.2* genes. On the other hand, *rom4.2* and *rom4.3* genes are separated by a 19 kb segment, where 11 *orfs* are encoded, only one of which corresponds to a 20S proteasome-related threonine protease (XP_001610115). In addition, immediately upstream of *rom4.5* lies a gene encoding ubiquitin fusion degradation protein (XP_001610110), also associated with proteasome function. *Rom4.2* is encoded in the opposite strand to the remaining *rom4* gene paralogs (Figure 1). No other rhomboid genes are encoded in chr 2.

Interestingly, the intergenic regions (IGs) upstream of the three most closely similar and tandemly arranged genes, *rom4.3*, *4.4* and *4.5*, also display a high degree of overall sequence identity. The IGs between *rom4.4* and *rom4.5* are 87% identical, and show 52% and 47% sequence identity with the IG upstream of *rom4.3*, respectively. Since the *rom4.3* upstream IG is shorter (467 bp) than the other two (747 bp each), 467 bp fragments conserved in the 5′ regions of *rom4.4* and *4.5* IGs were compared to *rom4.3* IG. Levels of identity were 83% (*rom4.4* vs. *rom4.5* IGs), 75% (*rom4.3* vs. *rom4.5* IGs) and 80% (*rom4.4* vs. *rom4.5* IGs). A highly polymorphic, roughly 120 bp-long segment starts 514 bp upstream of the start codon of *rom4.3* and 231 bp upstream of the start codons of *rom4.4* and *4.5* (Supplementary Figure S2).

### 2.2. In Silico Characterization of ROM4 Proteins

**Figure 2:**
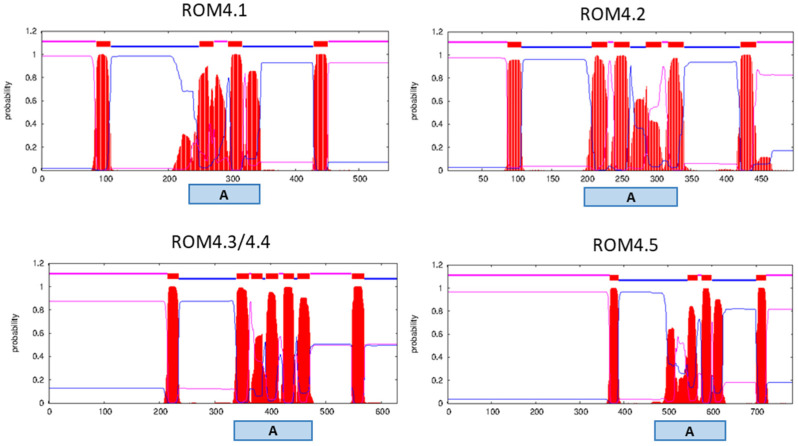
Predicted topology of *Babesia bovis* ROM4 paralogs. Purple, blue and red lines on top of each graph correspond to protein regions predicted as facing the extracellular milieu, the cytoplasm or inserted in the membrane, respectively. A: active site region. ROM4.3 and 4.4 are displayed in a single graph because their amino acid sequences are identical.

Comparison of the predicted amino acid (aa) sequences of the five *B. bovis* T2Bo ROM4 paralogs showed that ROM4.3 and 4.4 encode almost identical protein sequences, which display higher levels of identity with ROM4.5 than with the remaining two ROMs (Table 2, Figure S1). The highest levels of similarity among the five paralogs were found in the C-terminal half, corresponding to the region of TMDs (Supplementary Figure S1, Table 1, Figure 2). No sequence similarities exist between *rom4* genes and the other protease-related genes that lie in close proximity to them in the genome. 

Each *B. bovis* ROM4 protein contains 5–7 predicted TMDs. In addition, these proteins present hydrophilic regions in the N and C termini. In all cases, the active site lies in the region of TMDs, within the rhomboid family domain (cl21536) (Figure 2). ROMs do not possess a canonical signal peptide in their N-termini. 

**Figure 3:**
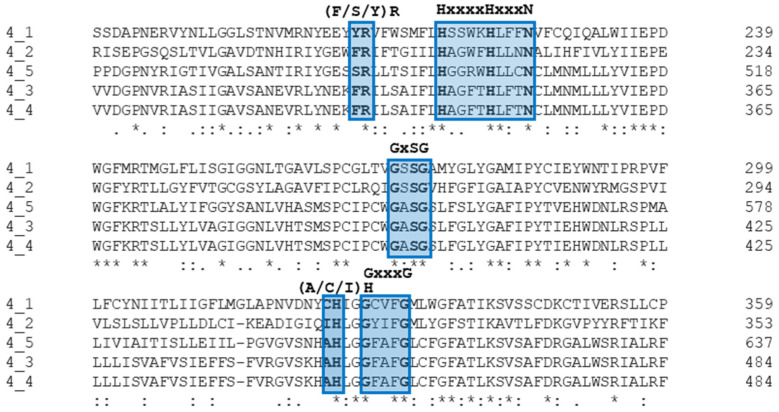
Alignment of the region, including the catalytic active site of *Babesia bovis* ROM4 paralogs showing the ROM-specific signature motifs of functional importance. Positions with fully conserved residues, residues that share strongly similar properties and residues that share weakly similar properties are marked with an asterisk, a colon and a period, respectively.

Importantly, all *B. bovis* ROM4 paralogs are predicted to be active and bear the conserved functional aa signatures corresponding to these families of proteins: (Y/F)R in Loop 1, HxxxN in TMD2, GxSG in TMD4 and (A/C/I/S)H and GxxxG in TMD6 (Figure 3) [16]. However, ROM4.2 has been wrongly annotated in the MEROPS database as a non-protease homologue, likely because the analysis was carried out on an incomplete *orf*. This has been recently revised and corrected [17]. 

### 2.3. Synteny Analysis of B. bovis rom4 Paralogs

We analyzed the syntenic relationships of *rom4* gene paralogs between different piroplasmid lineages (Table 3). The *rom4.1* gene, the ortholog to *P. falciparum rom4*, has syntenic counterparts in *Babesia* sensu stricto (s.s.), *Babesia* sensu lato (s.l.), *Theileria* s.s., *T. equi* and *Cytauxzoon felis*. Syntenic paralogs to *rom4.2* could only be found in *B. divergens* and *Babesia* sp. Xinjiang, both of which represent s.s. *Babesia*. Interestingly, *rom4.4* and *4.5* are jointly syntenic to a single gene in other *Babesia* s.s. species, a situation referred to as co-orthology. With the exception of *rom4.1*, no synteny was found for the remaining *B. bovis rom4* genes for *Babesia* s.l., *Theileria* s.s., *T. equi* or *C. felis*. 

### 2.4. Expression of Two rom4 Genes Is Significantly Increased in Xanthurenic Acid-Induced Xexual Stages of B. bovis Compared with Non-Induced Merozoite Suspensions

*In vitro* cultured *B. bovis* T2Bo merozoites were exposed to xanthurenic acid at 26 °C for 24 h (a) or incubated for the same time under normal culture conditions in the absence of this reagent (b). At the end of the incubation period, parasites were observed outside or inside of erythrocytes in (a) and (b), consistent with the presence of SS and AS, respectively. 

Subsequently, quantitative PCR protocols were set up to analyze the transcription levels of these gene paralogs in SS compared to AS. First, a housekeeping gene was selected as a control for data normalization, and melting curve analysis and efficiency tests were carried out. Two primer pairs (topo 1F/R and topo 2F/R) targeting *topoisomerase* (BBOV_III004820) and one pair of primers each for *actin* (BBOV_IV009790) and *gadph* (BBOV_II002540) genes were selected. Plasmid DNA or SS and AS cDNA samples were used as templates (Supplementary Table S1). All primer pairs yielded efficiencies of >80% and >90% when tested with plasmids and cDNA, respectively, which are considered adequate (Supplementary Table S2). *Actin* primers showed low specificity, as evidenced by the appearance of double peaks in the melting curve analysis. On the other hand, *topo* and *gadph* primer sets yielded single peaks, showing high specificity when tested with both plasmid and cDNA templates (Supplementary Figure S3). However, the Ct values obtained for *gadph* primers were considerably lower in SS as compared to AS samples, whereas *topo* primers gave similar Ct values for both types of samples and thus yielded the best results among those tested as normalization control genes for qPCR. The *topo* 2 primer set was finally chosen, since it gave the lowest Ct results when tested with plasmid DNA (Supplementary Table S2). 

**Figure 4:**
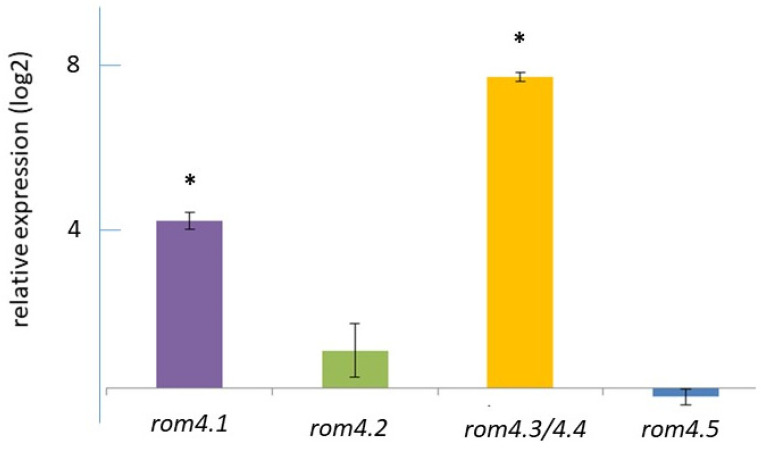
Relative transcription levels of *rom4* gene paralogs in xanthurenic acid-induced *Babesia bovis* cultures compared with non-induced cultures. Values were normalized based on the transcription level of *topoisomerase* as a control gene. Data are shown as the averages ± SD of triplicate determinations, and are representative of two independent experiments. (*) *p* < 0.01.

Transcript levels of *rom4.1*, *rom4.2*, *rom4.3/4.4* and *rom4.5* were evaluated by qPCR using cDNA obtained from SS and AS parasite suspensions. Transcription of all genes was verified in both types of preparations. Data were normalized with respect to the transcription level of *topoisomerase* and compared. The transcript levels of *rom4.1* and *rom4.3/4.4* were increased 18 and 218-fold in SS compared to AS (*p* < 0.01), respectively (relative SS/AS log2 ratio of 4.17 and 7.77). In addition, a non-significantly increased transcription level (*p* > 0.05) in SS compared to AS was observed for *rom4.2* (1.9-fold). Finally, *rom4.5* showed non-significant differences (*p* > 0.05) in AS as compared to SS (Figure 4).

### 2.5. Sequence Conservation of ROM4 Paralogs among Distinct B. bovis Geographic Isolates

As a first step to investigate whether polymorphism in *B. bovis* ROM4 sequences could be found between geographic isolates, the amino acid (aa) sequence of each ROM4 paralog of the reference strain *B. bovis* T2Bo available in Genbank was compared with the experimentally analyzed corresponding ROM4 sequences of the *B. bovis* R1A vaccine strain from Argentina. For both ROM4.1 and ROM4.2, peptide sequences showed 100% identity between these two strains. The *rom4.3* and *4.4* gene paralogs were amplified and analyzed together, since they showed identical aa sequences (Table 2). In this case, identity of 93% corresponding to a similarity of 97% was found for ROM4.3/4.4 between strains T2Bo and R1A. 

**Figure 5:**
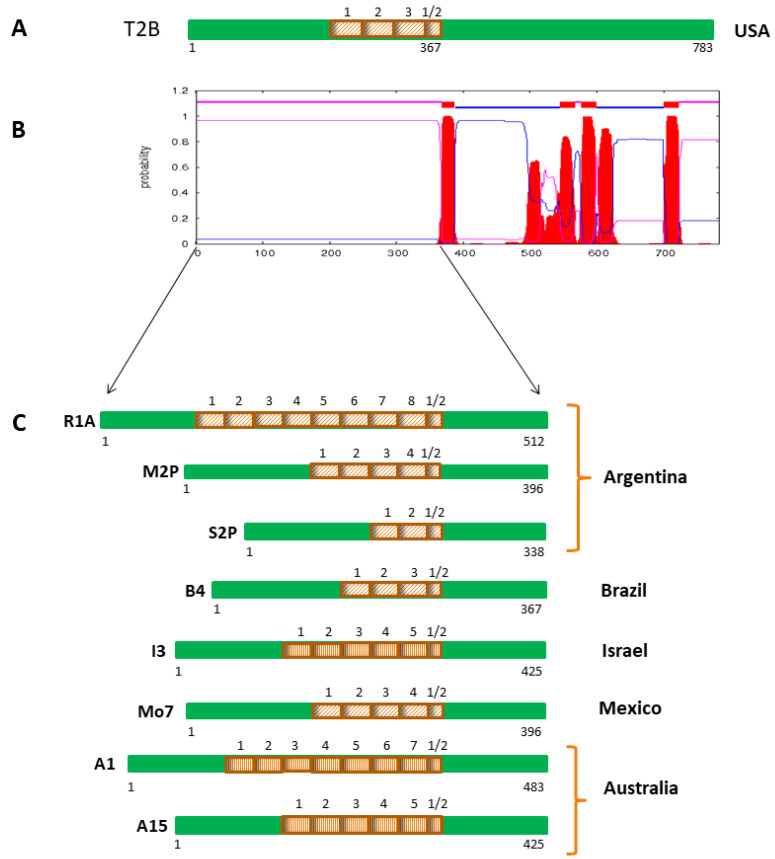
Schematic representation of a tandem repeat sequence found in ROM4.5 proteins of diverse *Babesia bovis* geographical isolates. (**A**) Scheme of whole length *B. bovis* ROM4.5 of the reference sequenced strain T2Bo from the USA, showing the positions and number of repeats. (**B**) Secondary structure of ROM4.5 of *B. bovis* T2Bo, showing the region that was sequenced in geographic isolates of diverse origin. (**C**) Schemes of amino acid sequences of the hydrophilic extracellular regions of ROM4.5 alleles from geographical isolates of *B. bovis*, indicating the positions and number of repeats. Green: 100% conservation among isolates; light brown boxes: perfect 29 aa tandem repeats present in different copy numbers in isolates from North America (T2Bo, Mo7), Brazil (B4), South Africa (SA2) and Argentina (R1A, M2P, and S2P); dark brown boxes: imperfect 29 aa tandem repeats found in different copy numbers in isolates from Australia (A1 and A15) and Israel (I3). Smaller boxes with a ½ sign correspond to truncated repeats.

Notably, in the case of ROM4.5, polymorphisms between T2Bo and R1A were exclusively found in the N-terminal half of the protein, which is predicted as a hydrophilic surface-exposed region (Supplementary Figure S5). This region contains a highly conserved 29 aa sequence that is 3.5 and 8.5 times tandemly repeated in the strains T2Bo and R1A, respectively (Figure 5, Supplementary Figure S5). In a second step, the N-terminal half of ROM4.5 from eight additional geographical isolates of *B. bovis* from Argentina, Mexico, South Africa, Israel and Australia were analyzed. This revealed a different number of tandem repeats of the 29 aa repeat sequence in each strain (Figure 5).

**Figure 6:**
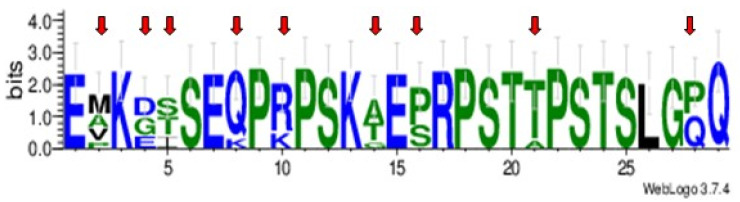
Sequence logo of an alignment of the 29 aa repeat sequences in ROM4.5 alleles of *Babesia bovis* isolates of different geographic origins. The alignment was generated using the twelve 29 aa slightly different repeats identified in *B. bovis* isolates originating from North and South America, South Africa, Australia and Israel. Red arrows mark nine polymorphic positions found in the analyzed sequences. The overall height of each position gives the grade of sequence conservation in bits, and the height of each aa in a given stack corresponds with its relative frequency. Hydrophilic aa are shown in blue; neutral aa are shown in green; and hydrophobic aa are shown in black.

The 29 aa repeat sequence was found to be 100% conserved in isolates from North and South America and South Africa, resulting in perfect tandem repeats. In contrast, imperfect tandem repeats were observed in the Israeli I3 strain, which had four slightly different variants of the 29 aa repeat sequence, and in the A1 and A15 strains from Australia, which had three and four different 29 aa repeat sequence variants, respectively. When all 29 aa repeat sequence variants were aligned and compared, nine positions were found to be polymorphic (Figure 6, Supplementary Figure S6). In all analyzed strains, the repeat region is flanked by the conserved aa motifs SVSGVSK and SKLST (Supplementary Figures S5 and S7). 

**Figure 7:**
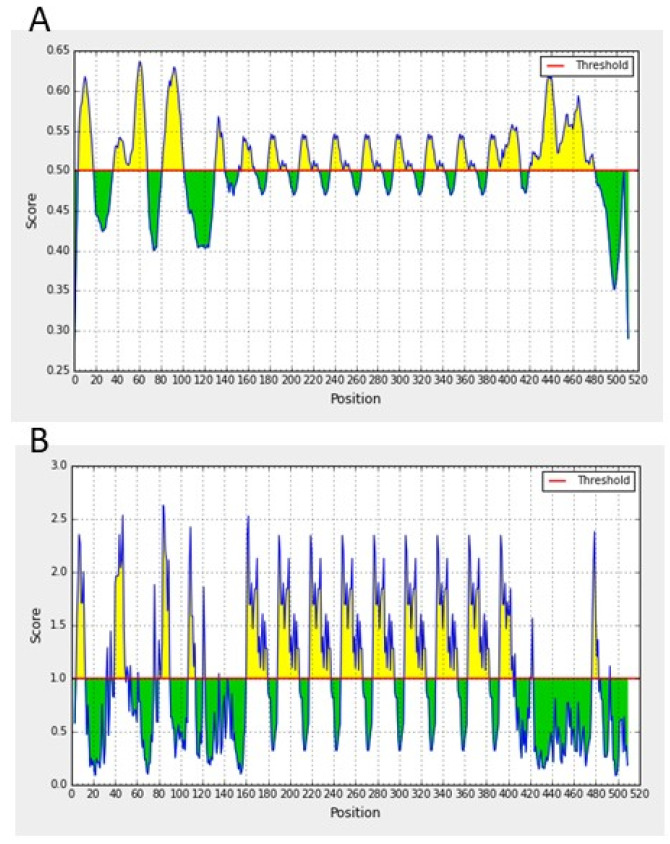
Prediction of B-cell epitopes (**A**) and surface accessibility (**B**) in the N-terminal hydrophilic region (aa 1–512) of ROM4.5 of the *Babesia bovis* Argentine R1A strain. Prediction of a B-cell epitope with high surface accessibility (yellow peaks) falls within the repetitive region.

Amino acids in the repeat sequence are mostly hydrophilic or neutral (Figure 6). Noteworthily, the repeat region contains a B-cell epitope, predicted by all used programs, with high surface accessibility, as illustrated in Figure 7 for the Argentine R1A strain of *B. bovis*. Despite observed moderate sequence polymorphisms, the corresponding amino acid regions in the Israeli and Australian strains are also predicted to have high antigenicity. 

The average ratio of non-synonymous over synonymous substitutions (dN/dS) was calculated for the repeat region, using the nucleotide sequences encoding the different repeat variants found among *B. bovis* geographical isolates: (*n* = 1 for American isolates, *n* = 1 for the South African isolate, *n* = 7 for the two Australian isolates and *n* = 4 for the Israeli isolate). An average dN/dS value of 0.24 was obtained, strongly indicative of negative selection (Supplementary Figure S6). 

Apart from the diversity in the number of repetitive sequences and the slight differences found in the repeat sequence of Israel and Australia, the remainder of the N-terminal regions had little or no polymorphisms. As an example, Supplementary Figure S6 shows an alignment of this region between *B. bovis* Mo7 from Mexico and A15 from Australia, two geographic isolates of very distant origins.

## 3. Discussion

ROMs were first discovered in *Drosophila melanogaster*, in which they were shown to be involved in the epidermal growth receptor signaling pathway. Soon after, ROM representatives were found in all kingdoms of life, and recognized as part of an ancient, large family of intramembrane proteases containing members with and without protease catalytic activity [14,15,18]. The molecular mechanisms and functions where ROMs are involved are diverse, ranging from cell signaling in animals to quorum sensing in bacteria, and from flower development in plants to host cell invasion in apicomplexan protozoa [11,19]. In Apicomplexa, research on ROMs was first carried out in *T. gondii*, where five different members that received the denomination ROM1 to ROM6 were described. ROM4 and 5 are paralogs and both belong to the ROM4 family (sometimes referred to as ROM4/5), whereas ROM2 is confined to *T. gondii*. It was later observed that the ROM repertoire varies considerably among members of the Apicomplexa phylum [12]. Indeed, it has been found that *P. falciparum* has not only orthologs of *T. gondii* ROM1, 3, 4/5 and 6, but contains four additional members of this family, which received the denomination ROM7 to 10 [20]. Moreover, piroplasmids share with *P. falciparum* the presence of ROM4, 6, 7 and 8, but ROM1, 3, 9 or 10 are absent in the first. Notably, while *P. falciparum* has only one ROM4 member, piroplasmids have an expanded number of two to five ROM4 paralogs, with *B. bovis* showing the highest number of five paralogs among the studied organisms [16]. 

As mentioned before, the ROM4 of *P. falciparum* and *T. gondii* have been demonstrated to cleave adhesins on the parasite surface, a prerequisite to allowing parasite internalization into the host cell [13,14]. The release of two such adhesins, AMA-1 and TRAP, from the parasite surface into the extracellular medium, has been verified in *B. bovis* in vitro cultures, but the protease involved in this cleavage has not yet been identified [21,22]. We hypothesize that of the five *B. bovis* ROM4 paralogs, ROM4.1 exerts this role, since it is the ortholog of ROM4 of *P. falciparum* and *T. gondii* [16].

The particular expansion of the *rom4* locus to a gene family of five paralogs in *B. bovis* is intriguing. The fact that all are transcriptionally active in AS and SS and in bovine and tick stages suggests their functional relevance. It remains to be explored if these five paralogs exert different roles associated with the interaction of the tick and mammalian host cell. The tandemly arranged *rom4.3*, *4.4* and *4.5* have a single syntenic counterpart in other *Babesia* s.s. parasites which, together with their high sequence similarity, suggests they have expanded recently into this *rom4* subgroup in *B. bovis*, representing true in-paralogs. Importantly, the presented synteny data confirm the orthology results obtained by bidirectional best hit (BBH) using BLAST and phylogenetic analysis in a previous study [16]. Given that the only *rom4* paralog with syntenic counterparts in all piroplasmids is *rom4.1*, we can speculate that this corresponds to the ancestral rhomboid 4 gene of *B. bovis*, which, through duplications and subsequent sequence divergence, gave rise to the currently existing paralogs. Likely, the tandemly arranged *rom4.3*, *4.4* and *4.5* originated from the most recent of these duplication events. 

In the present study, we show that *rom4.1* and *rom4.3/4.4* transcription levels are significantly increased in xanthurenic acid-induced parasite suspensions as compared to non-induced ones. Xanthurenic acid is a metabolite present in the gut of mosquitoes that induces gametogenesis in *P. falciparum* [23,24]. Correspondingly, incubation of erythrocyte suspensions infected with *B. bigemina*, *B. bovis* or *B. ovata* with this compound has been shown to yield extracellular suspensions of parasites, which are morphologically different from merozoites and express sexual stage-specific markers. These parasite stages are considered to represent haploid gametocytes and diploid zygotes. Accordingly, xanthurenic acid-induction protocols have been used to study the life cycle of *Babesia* spp. and antigen expression patterns in SS [25,26,27,28]. 

The patterns of *rom4* expression in xanthurenic induced SS and non-induced AS cultures observed by RT-qPCR showed some agreement with those in tick and merozoite stages observed by RNAseq in the study of Ueti et al. [17]. *B. bovis* tick stages show a dramatic increase in transcription of *rom4.1* of 1.3 × 10^4^-fold as compared to bovine stages, and in the present study, we observed the same tendency but a more modest increase in 18-fold in the transcription of this gene, analyzed by RT-qPCR in xanthurenic acid-induced SS as compared to non-induced AS. It is possible to hypothesize that certain processes in which ROM4.1 is involved and that are critical for the parasitic stages that take place in the tick milieu, such as gamete formation and fusion, are elicited in vitro by exposure to xanthurenic acid. Additionally, in the case of *rom4.5*, expression was increased 14-fold in asexual bovine stages as compared to sexual tick stages [17]. In our study, a 1.8-fold increase was observed in AS, as compared to SS, although this difference was non-significant (*p* > 0.05). In vivo and in vitro results of the expression of the other *rom4* paralogs did not correlate. Our study showed a significant increase in *rom4.3/4.4* transcription levels in SS as compared to AS. However, *rom4.4* was found to be upregulated in bovine stages compared to tick stages by RNAseq. No significant differences were found for *rom4.3*. Finally, tick stages showed upregulation of *rom4.2*, though no significant differences in transcription of this gene were found in our study between AS and SS [17]. Our study indicates that it is not possible to directly extrapolate the results of the xanthurenic acid-induction studies to the complex events that take place in the tick environment, although they are sometimes useful as an indication of which antigens are relevant for sexual parasitic stages. Importantly, *Babesia* sp. s.s. parasite stages present in tick hemolymph are likely dominated by kinetes, with activated mechanisms for tick cell invasion and asexual replication [4]. Xanthurenic acid-induced *B. bovis* parasite suspensions, on the other hand, are characterized by the presence of gamete-like forms with projections and surface ruffles, which can fuse, forming multinucleated syncytia. Completion of kinete formation with nuclear fusion, which is assumed to be eventually followed by meiosis, has not been observed in this type of suspensions so far [26]. These different moments of parasitic life surely have specific processes where ROM proteins are involved. Future functional studies with available gene editing tools for *B. bovis* will help to elucidate the functional relevance of ROM4 paralogs in the parasite life cycle [9]. 

It is interesting to note that a differential regulation was observed for in vivo and in vitro AS and SS for the tandemly arranged *rom4.3*, *rom4.4* and *rom4.5* genes in spite of overall high sequence identity among their upstream intergenic regions. However, the presence of a distinctive, ~120 bp polymorphic region could be connected to gene-specific regulatory protein binding sites. Other mechanisms that guarantee the specific expression control of each of these genes, such as enhancers, cannot be ruled out. 

Notably, ROM4.5 contains a highly conserved 29 aa sequence bearing a predicted B-cell epitope expanded to up to eight repeats in the analyzed strains, in a hydrophilic, surface-exposed and accessible region of the protein. We thus hypothesized that this region is subject to positive selection pressure of the host immune system. To test this hypothesis, we analyzed the average ratio of the number of non-synonymous substitutions per non-synonymous site to the number of synonymous substitutions per synonymous site, as an indication of selective pressure exerted on the coding sequence of the repetitive region. An average dN/dS value of 0.24 was estimated, which does not support the hypothesis of the existence of positive selection in this region. However, analysis of a larger number of isolates would be important to strengthen this conclusion. 

It can be speculated that the repeats region acts as a decoy for the host immune defense system, diverting the responses against other functionally relevant molecules required for invasion of the host cell by the parasite, as has been suggested for surface antigens reported in *B. bovis* and other piroplasmid species [29,30]. However, the immunogenicity of this region needs experimental confirmation to substantiate this hypothesis. Noteworthily, polymorphisms observed among *rom4.5* alleles might provide a tool for strain genotypification. 

As for other protease-coding genes, transcription of *rom4* paralogs in the bovine asexual stage of *B. bovis* did not differ significantly between the virulent T2Bo strain and an attenuated strain derived from it by serial blood passages in splenectomized cattle. This indicates that the pathogenic/attenuated phenotype does not depend on a differential transcription of these genes [31]. 

In addition to ROM4, ROM6, 7 and 8 have been described in *B. bovis* and in all other piroplasmids analyzed [16]. *B. bovis* ROM6 (XP_001609020) is the only piroplasmid rhomboid not exclusive to apicomplexans. It is encoded in chromosome 1 (chr1); has mitochondrial localization; and participates in various physiological processes within this organelle, including apoptosis, electron transport chain and homeostasis [32]. No function has yet been assigned to the catalytically active ROM7 (XP_001611188), encoded in chr3 of *B. bovis*; or the inactive ROM8 (XP_001610508), encoded in chr4 of *B. bovis*. Since their presence is shared by *Plasmodium* spp. and piroplasmids, their function may be related to intraerythrocytic processes taking place in the Aconoidasida, to which both groups of protozoa belong [16]. In addition to the mentioned ROMs, *B. bovis* possesses two members of the “derlin” subfamily, XP_001612151 and XP_001611988, encoded in chr4 (Florin-Christensen, M. and Schnittger, L., unpublished observations). This type of catalytically inactive rhomboid was discovered in yeasts, and later found to be represented in many different lower and higher eukaryotes. Their function appears to be related to retrotranslocation of misfolded proteins from the endoplasmic reticulum to the cytoplasm, where they are ubiquitinated and degraded in the proteasome [33].

Currently, the only available vaccines against *B. bovis* are based on suspensions of erythrocytes infected with live attenuated parasites. Although generally effective and safe in younger animals, these vaccines can cause disease in adult cattle, can co-transmit other pathogens and have a cumbersome production process. Thus, the search for alternative vaccination strategies and formulations is an active field of research. A substantial amount of information about *B. bovis* antigens expressed in the vertebrate stage that could serve in vaccine formulations against sporozoites and merozoites has accumulated during the last two or three decades. The search for possible antigens has mostly focused on proteins that fulfill relevant roles in host–pathogen interactions of the merozoite stage, which are able to elicit an adequate humoral and cellular immune response in the bovine host [1,34]. On the other hand, information about antigen expression in tick stages has lagged behind [9]. However, recent in vivo and in vitro expression analyses of individual genes and transcriptomic studies are rapidly filling this gap. Antigens that are particularly relevant for the parasite developmental stages in the tick are attractive candidates for transmission-blocking vaccines [9,25,26,35,36,37,38]. Antigens that fulfill essential roles both for the intraerythrocytic merozoite stage and for tick stages, as might be the case for ROM4.1, for example, could be of particular interest for the development of vaccine formulations to block parasite growth in the bovine and block transmission by the tick. Further studies to elucidate the role(s) fulfilled by this protein are needed to furnish this speculation. 

Importantly, ROM4s of other Apicomplexan parasites have shown potential as vaccine candidates. Indeed, vaccination of chicken with a recombinant form of *Eimeria tenella* ROM4 elicited a cellular and humoral response and high protection levels against challenge with the homologous *E. tenella* strain [39]. Similarly, mice vaccinated with a plasmid encoding *T. gondii* ROM4, or with a combined vaccination scheme of *T. gondii rom4*-DNA plasmid followed by a ROM4 peptide, showed humoral and cellular responses, increased survival rates and fewer brain cysts upon challenge with a virulent *T. gondii* strain [40,41]. Since ROM functional domains are not surface-exposed, the mode of action of these ROM-based vaccines is intriguing. It might be hypothesized that antibody blockading of surface regions relevant for their catalytic function results in impaired parasite growth, but this needs experimental confirmation.

ROMs are also considered attractive targets for chemotherapeutic measures against different pathogens, and several ROM-specific inhibitors have been developed [42,43,44] Two of them, rhomboid-inhibiting ketoamide (RiKa) and rhomboid-inhibiting boronate (RiBn), were recently shown to specifically hamper in vitro *P. falciparum* invasion of erythrocytes [45]. 

In summary, this study presents new information regarding the structures, transcription levels and sequence polymorphisms of ROM4 paralogs and their products in *B. bovis,* suggesting several possible roles in host–pathogen interactions. This knowledge may facilitate the development of novel control tools against bovine babesiosis and other piroplasmid-caused diseases of domestic animals and humans.

## 4. Materials and Methods

### 4.1. In Silico Analysis

Locations, lengths and introns of *rom4* paralogs, and the presence of other protease-coding genes in the proximity of *rom4* genes, were analyzed in the NCBI website (www.ncbi.nlm.nih.gov/, accessed on 15 October 2021) for the sequenced *B. bovis* T2Bo strain [46]. Transmembrane domains were predicted using the TMHMM algorithm (services.healthtech.dtu.dk/service.php?TMHMM-2.0), and the presence of a signal peptide was investigated with SignalP 5.0 (services.healthtech.dtu.dk/service.php?SignalP-5.0). Discovering the location of the active site and prediction of catalytic activity were carried out in the MEROPS server (www.ebi.ac.uk/merops/). Multiple sequence alignments were performed by Clustal omega (www.ebi.ac.uk/Tools/msa/clustalo/). Percentages of identity and similarity were calculated at the SIAS website (imed.med.ucm.es/Tools/sias.html). Synteny analysis was carried out at Piroplasma DB (piroplasmadb.org/piro/app). Conservation of amino acids in the repeat sequences was visualized using Weblogo (weblogo.berkeley.edu/logo.cgi). B-cell epitopes were predicted in the following web servers: ABCpred (crdd.osdd.net/raghava//abcpred/); Emboss Antigenic server (www.bioinformatics.nl/cgi-bin/emboss/antigenic); Bepipred Linear Epitope prediction 2.0 (tools.immuneepitope.org/bcell/). Accessibility was evaluated with the Emini Surface Accessibility Prediction (tools.immuneepitope.org/bcell/). The average ratio of non-synonymous vs. synonymous substitutions (dN/dS) in the repeat region variants was calculated with SNAP v2.1.1. (hcv.lanl.gov/content/sequence/SNAP/SNAP.html).

### 4.2. Production of cDNA of In Vitro Cultured AS and SS

*B. bovis* T2Bo strain merozoites were in vitro propagated in microaerophilic stationary phase (MASP) culture following established procedures [38]. Infected erythrocytes were harvested by centrifugation, suspended in a solution of HL-1 medium, bovine serum, antibiotic and antimycotic, and incubated with 100 μM xanthurenic acid for 24 h at 26 °C in air, or without xanthurenic acid in a CO_2_ incubator at 37 °C, to obtain SS or AS, respectively, as described in [25]. Suspensions were then harvested by centrifugation and the pellets suspended in RNAlater (Ambion) and kept at −20 °C until use. Total RNA was extracted from both types of cultures using RNAqueous^®^ Kit (Ambion), following the manufacturer’s recommendations and quantified in a NanoDrop (ThermoScientific, Waltham, MA, USA) equipment. The removal of genomic DNA was confirmed by RT-PCR targeting a 259 bp fragment of *rom4.3/4.4* using primers F: TCAGCCAGAGCAACCCGA and R: GCCTATGAATGAAAGTGTC, with and without addition of reverse transcriptase, and with AS and SS RNA as template, following standard conditions (Supplementary Figure S7). cDNA was synthesized from 150 ng total RNA of each sample with the Superscript^®^ First-strand cDNA synthesis kit (Invitrogen, Waltham, MA, USA) following the manufacturer’s recommendations. 

### 4.3. Transcriptional Analysis 

Specific oligonucleotides to be used in qPCR for each *B. bovis* T2Bo *rom4* paralog, and for the housekeeping genes coding for topoisomerase II (BBOV_III004820), actin (BBOV_IV009790) and gadph (BBOV_II002540), were designed using the PrimerExpress 2.0 software (Applied Biosystems), considering the following parameters: a length of 20–22 nt, an amplicon size of ~60–240 bp, a hybridization temperature between 50 and 60 °C, ~50% GC content and a hybridization target within gene exons (Supplementary Table S1). 

Recombinant plasmids containing each target sequence were prepared to be used in the qPCR set-up. To this end, the corresponding DNA fragments were amplified by direct PCR using DNA from the T2Bo as template in a final volume of 25 μL containing 2.5 mM of each dNTP, 20 μM of each primer, 2.5 U PFU polymerase (Thermo Scientific, Waltham, MA, USA) with its corresponding buffer and 6.6 ng DNA as template or water as negative control. The cycling conditions consisted in a denaturing period at 95 °C for 3 min; 35 cycles of denaturing at 95 °C for 30 s, hybridization at 58 °C for 30 s and extension at 72 °C for 1 min; and a final extension period at 72 °C for 10 min. Amplicons were visualized in ethidium bromide-containing 1% agarose gels and band sizes were assessed by comparison with a 1 kb Plus DNA marker (Invitrogen). Amplicons were cloned in CloneJet (Thermo Fisher Scientific), and recombinant plasmids were used to transform competent *E. coli* DH5α cells. After plating in ampicillin-containing LB-agar plates, colonies containing recombinant plasmids were selected by colony PCR and grown overnight in LB-ampicillin. Plasmids were purified from cultures using a DNA Puriprep P-kit (Inbio Highway, Argentina) and quantified spectrophotometrically. 

qPCR reactions were carried out using the SYBR Green qPCR Master Mix (Thermo Scientific) in a final volume of 10 μL, containing 0.2 μM of each primer, 1 μL of cDNA or plasmid template and 0.2 μL *ROXdye* (Thermo Scientific) as a passive reference control to normalize PCR-unrelated fluorescence variations. Each reaction was run in parallel with a negative control containing 1 μL ultrapure water instead of DNA. Amplification was carried out in a StepOne Plus thermocycler (Applied Biosystems) with a cycling protocol of 5 min at 95 °C, followed by 40 cycles of 95 °C for 1 min, 55 °C for 30 s and 72 °C for 40 s. The qPCR protocol was designed following MIQE international rules [47]. Primer efficiency and Cts were evaluated using the LinRegPCR software [48]. The specificity of each reaction was analyzed by melting curve analysis using the StepOne Software v2.3 software incorporated in the StepOne Plus thermocycler (Applied Biosystems, Waltham, MA, USA).

Once the primer efficiency and reaction specificity had been verified and a housekeeping gene target had been chosen (see Section 2.4; Supplementary Table S2), transcription levels were determined in three biological replicas of AS and SS cultures, each of which was evaluated in triplicate qPCR reactions. Ct data were normalized to the transcription levels of the chosen housekeeping gene target qPCR values using the fgStatistics software. The same software was used to calculate the statistical significance of the data (*p* < 0.05) [49,50]. 

### 4.4. DNA Samples 

DNA samples of *B. bovis* geographical isolates from Argentina (R1A, S2P, M2P), Brazil (B4), Mexico (Mo7), Australia (A1 and A15), Israel (I3) and South Africa (SA2) were used as templates for PCR amplification of *rom4* alleles. Data on these strains are included in Supplementary Table S3. 

### 4.5. Sequence Analysis of rom4 Alleles

Alleles of *rom4* paralogs from different *B. bovis* geographical isolates were amplified by PCR, cloned and sequenced for polymorphism analysis. The whole sequences of *rom4.1*, *rom4.2*, *rom4.3/4.4* and *rom4.5* of the R1A Argentine vaccine strain; and the N-terminal hydrophilic-coding sequences of *rom4.5* from different geographical isolates were analyzed. Primers were designed to amplify overlapping segments of ~500–1500 bp for each gene (Supplementary Table S4; Supplementary Figure S8). PCR amplification, cloning and plasmid purification were essentially carried out as mentioned in Section 4.3, adjusting the hybridization temperature in the PCR to each primer pair. Sequencing of both strands was carried out using the Sequencing Service of the National Institute of Agricultural Technology (INTA, Argentina) or Macrogen (South Korea). Sequences were edited using BioEdit and deposited in the GenBank under Accession numbers OM320986 to OM320997.

## Figures and Tables

**Table 1:** *Babesia bovis* ROM4 paralogs.

Proposed Designation	Protein ID	Size (aa)	MEROPS ID	Active Site (aa)	Gene Locus
ROM4.1	EDO06560.2 *	547	MER1081995	238–341	BBOV_II006100
ROM4.2	EDO06557.2 *	498	MER1079457	193–330	BBOV_II006070
ROM4.3	XP_001610113	629	MER0112671	308–486	BBOV_II005950
ROM4.4	XP_001610112	641			BBOV_II005940
ROM4.5	XP_001610111	783	MER1075434	468–621	BBOV_II005930

(*) Protein sequences re-annotated as described in [17].

**Table 2:** Percentages of identity/similarity between pairs of ROM4 protein paralogs.

	ROM4.1	ROM4.2	ROM4.3	ROM4.4
ROM4.2	26.9/37.3	-	-	-
ROM4.3	25.6/36.2	33.1/45.4	-	-
ROM4.4	25.8/36.4	33.3/45.6	100/100	-
ROM4.5	25.9/35.6	33.1/44.8	73.9/78.7	72.5/77.2

**Table 3:** Synteny analysis of *rom4* gene paralogs of *B. bovis* with corresponding orthologs in other piroplasmid lineages (clade designation is given according to Schnittger et al. [2].

Phylogenetic Placement	Piroplasmid Species	*B. bovis rom4* Gene Paralogs				
		*rom4.1*	*rom4.2*	*rom4.3*	*rom4.4*	*rom4.5*
*Babesia s.s.* (Clade VI)	*B. bigemina*	BBBOND_0104040	-	-	BBBOND_0104210	
	*B. ovata*	BOVATA_022110	-	-	BOVATA_021930	
	*B. divergens*	Bdiv_004020c	Bdiv_003990	-	Bdiv_003860c	
	*Babesia* sp. Xinjiang	BXIN_2807	BXIN_2802	-	BXIN_2789	
*Babesia* s.l. (Clade I)	*B. microti*	BMR1_02g02777	-	-	-	-
*Theileria* s.s. (Clade V)	*T. annulata*	TA13905	-	-	-	-
	*T. orientalis*	TOT_020000558	-	-	-	-
	*T. parva*	TpMuguga_02g02170	-	-	-	-
*Theileria* s.l. (Clade IV)	*T. equi*	BEWA_023690	-	-	-	-
*Cytauxzoon* (Clade IIIb)	*C. felis*	CF003707	-	-	-	-

## Data Availability

*Rom4* gene sequences have been deposited in the GenBank (accession numbers OM320986 to OM320997).

## Supplementary Materials

The following supporting information can be downloaded at: https://www.mdpi.com/article/10.3390/pathogens11030344/s1, Table S1. Oligonucleotides used in qRT-PCR; Table S2. Ct and efficiency values obtained by qPCR targeting potential reference genes of *Babesia bovis*; Table S3. Information on the *Babesia bovis* strains used to study *rom4* sequence polymorphism; Table S4. Primers used to amplify *rom4* segments for polymorphism studies; Figure S1. Clustal omega alignment of ROM4 paralogs of *Babesia bovis*, T2Bo strain; Figure S2. Clustal omega alignment of intergenic regions upstream of *rom4.3* (Int3), *rom4.4* (Int4) and *rom4.5* (Int5); Figure S3. Melting curve analysis to select an adequate housekeeping gene as normalization control of transcription in *Babesia bovis*, as determined by qPCR; Figure S4. Clustal omega alignment of nucleotide sequences of *rom4.5* alleles of *Babesia bovis* T2B and R1A; Figure S5. Clustal omega alignment of the N-terminal region of ROM4.5 of *Babesia bovis* T2Bo and R1A; Figure S6. Analysis of polymorphism and synonymous and non-synonymous substitutions in the repeat region of ROM4.5; Figure S7. Clustal omega alignment between N-terminal regions of ROM4.5 of the Mexican Mo7 strain and the Australian A15 strain of *Babesia bovis*; Figure S8. PCR and RT-PCR amplification of *Babesia bovis rom4.3/4.4*; Figure S9. Scheme of PCR amplification of *rom4* alleles for polymorphism analysis. References [51,52,53,54,55,56,57,58] are cited in the Supplementary Materials.

## References

[1] Florin-Christensen M., Suarez C.E., Rodriguez A.E., Flores D.A., Schnittger L. (2014). Vaccines against bovine babesiosis: Where we are now and possible roads ahead. Parasitology.

[2] Schnittger L., Rodriguez A.E., Florin-Christensen M., Morrison D.A. (2012). *Babesia*: A world emerging. Infect. Genet. Evol..

[3] Schnittger L., Ganzinelli S., Bhoora R., Omondi D., Nijhof A.M., Florin-Christensen M. (2022). The Piroplasmida *Babesia*, *Cytauxzoon*, and *Theileria* in farm and companion animals: Species compilation, molecular phylogeny, and evolutionary insights. Parasitol. Res..

[4] Jalovecka M., Hajdusek O., Sojka D., Kopacek P., Malandrin L. (2018). The complexity of piroplasms life cycles. Front. Cell. Infect. Microbiol..

[5] Bock R., Jackson L., de Vos A., Jorgensen W. (2004). Babesiosis of cattle. Parasitology.

[6] Ganzinelli S., Rodriguez A.E., Schnittger L., Florin-Christensen M., Florin-Christensen M., Schnittger L. (2018). Babesia in domestic ruminants. Parasitic Protozoa of Farm Animals and Pets.

[7] Trueman K.F., McLennan M.W. (1987). Bovine abortion due to prenatal *Babesia bovis* infection. Aust. Vet. J..

[8] Florin-Christensen M., Schnittger L. (2009). Piroplasmids and ticks: A long-lasting intimate relationship. Front. Biosci..

[9] Suarez C.E., Alzan H.F., Silva M.G., Rathinasamy V., Poole W.A., Cooke B.M. (2019). Unravelling the cellular and molecular pathogenesis of bovine babesiosis: Is the sky the limit?. Int. J. Parasitol..

[10] Klemba M., Goldberg D.E. (2002). Biological roles of proteases in parasitic protozoa. Annu. Rev. Biochem..

[11] Freeman M. (2004). Proteolysis within the membrane: Rhomboids revealed. Nat. Rev. Mol. Cell Biol..

[12] Dowse T.J., Soldati D. (2005). Rhomboid-like Proteins in Apicomplexa: Phylogeny and Nomenclature. Trends Parasitol..

[13] Lin J., Meireles P., Prudêncio M., Engelmann S., Annoura T., Sajid M., Chevalley-Maurel S., Ramesar J., Nahar C., Avramut C.M.C. (2013). Loss-of-function analyses defines vital and redundarnt functions of the *Plasmodium* rhomboid protease family. Mol. Microbiol..

[14] Dogga S.K., Soldati-Favre D. (2016). Biology of Rhomboid Proteases in Infectious Diseases. Semin. Cell Dev. Biol..

[15] Düsterhöft S., Künzel U., Freeman M. (2017). Rhomboid Proteases in Human Disease: Mechanisms and Future Prospects. Biochim. Biophys. Acta.

[16] Gallenti R., Poklepovich T., Florin-Christensen M., Schnittger L. (2021). The repertoire of serine rhomboid proteases of piroplasmids of importance to animal and human health. Int. J. Parasitol..

[17] Ueti M.W., Johnson W.C., Kappmeyer L.S., Herndon D.R., Mousel M.R., Reif K.E., Taus N.S., Ifeonu O.O., Silva J.C., Suarez C.E. (2020). Transcriptome dataset of *Babesia bovis* life stages within vertebrate and invertebrate hosts. Data Brief.

[18] Koonin E.V., Makarova K.S., Rogozin I.B., Davidovic L., Letellier M.C., Pellegrini L. (2003). The rhomboids: A nearly ubiquitous family of intramembrane serine proteases that probably evolved by multiple ancient horizontal gene transfers. Genome Biol..

[19] Urban S., Freeman M. (2002). Intramembrane proteolysis controls diverse signaling pathways throughout evolution. Curr. Opin. Genet. Dev..

[20] Santos J.M., Graindorge A., Soldati-Favre D. (2012). New Insights into Parasite Rhomboid Proteases. Mol. Biochem. Parasitol..

[21] Gaffar F.R., Yatsuda A.P., Franssen F.F.J., De Vries E. (2004). Erythrocyte invasion by *Babesia bovis* merozoites is inhibited by polyclonal antisera directed against peptides derived from a homologue of *Plasmodium falciparum* apical membrane antigen 1. Infect. Immun..

[22] Gaffar F.R., Yatsuda A.P., Franssen F.F.J., De Vries E.A. (2004). *Babesia bovis* merozoite protein with a domain architecture highly similar to the thrombospondin-related anonymous protein (TRAP) present in *Plasmodium* sporozoites. Mol. Biochem. Parasitol..

[23] Billker O., Lindo V.S., Panico M., Etienne A., Paxton T., Dell A., Rogers M.C., E Sinden R., Morris H.R. (1998). Identification of xanthurenic acid as the putative inducer of malaria development in the mosquito. Nature.

[24] Garcia G.E., Wirtz R.A., Barr J.R., Woolfitt A., Rosenberg R. (1998). Xanthurenic acid induces gametogenesis in *Plasmodium*, the malaria parasite. J. Biol. Chem..

[25] Hussein H.E., Bastos R.G., Schneider D.A., Johnson W.C., Adham F.K., Davis W.C., Laughery J.M., Herndon D.R., Alzan H.F., Ueti M.W. (2017). The *Babesia bovis* hap2 gene is not required for blood stage replication, but expressed upon in vitro sexual stage induction. PLoS Negl. Trop. Dis..

[26] Hussein H.E., Johnson W.C., Taus N.S., Capelli-Peixoto J., Suarez C.E., Mousel M.R., Ueti M.W. (2021). Differential expression of calcium-dependent protein kinase 4, tubulin tyrosine ligase, and methyltransferase by xanthurenic acid-induced *Babesia bovis* sexual stages. Parasites Vectors.

[27] Mosqueda J., Falcon A., Alvarez A.J., Ramos A.J., Oropeza-Hernandez L.F., Figueroa J.V. (2004). *Babesia bigemina* sexual stages are induced in vitro and are specifically recognized by antibodies in the midgut of infected *Boophilus microplus* ticks. Int. J. Parasitol..

[28] Nguyen T.T., Dang-Trinh M.A., Higuchi L., Mosqueda J., Hakimi H., Asada M., Yamagishi J., Umemiya-Shirafuji R., Kawazu S.I. (2019). Initiated *Babesia ovata* Sexual Stages under In Vitro Conditions Were Recognized by Anti-CCp2 Antibodies, Showing Changes in the DNA Content by Imaging Flow Cytometry. Pathogens.

[29] Schnittger L., Katzer F., Biermann R., Shayan P., Boguslawski K., McKellar S., Beyer D., Shiels B.R., Ahmed J.S. (2002). Characterization of a polymorphic *Theileria annulata* surface protein (TaSP) closely related to PIM of *Theileria parva*: Implications for use in diagnostic tests and subunit vaccines. Mol. Biochem. Parasitol..

[30] Suarez C.E., Laughery J.M., Bastos R.G., Johnson W.C., Norimine J., Asenzo G., Brown W.C., Florin-Christensen M., Goff W.L. (2011). A novel neutralization sensitive and subdominant RAP-1-related antigen (RRA) is expressed by *Babesia bovis* merozoites. Parasitology.

[31] Mesplet M., Palmer G.H., Pedroni M.J., Echaide I., Florin-Christensen M., Schnittger L., Lau A.O. (2011). Genome-wide analysis of peptidase content and expression in a virulent and attenuated *Babesia bovis* strain pair. Mol. Biochem. Parasitol..

[32] Lysyk L., Brassard R., Touret N., Lemieux M.J. (2020). PARL Protease: A Glimpse at Intramembrane Proteolysis in the Inner Mitochondrial Membrane. J. Mol. Biol..

[33] Nejatfard A., Wauer N., Bhaduri S., Conn A., Gourkanti S., Singh N., Kuo T., Kandel R., Amaro R.E., Neal S.E. (2021). Derlin rhomboid pseudoproteases employ substrate engagement and lipid distortion to enable the retrotranslocation of ERAD membrane substrates. Cell Rep..

[34] Florin-Christensen M., Schnittger L., Bastos R.G., Rathinasamy V.A., Cooke B.M., Alzan H.F., Suarez C.E. (2021). Pursuing effective vaccines against cattle diseases caused by apicomplexan protozoa. CAB Rev..

[35] Bastos R.G., Suarez C.E., Laughery J.M., Johnson W.C., Ueti M.W., Knowles D.P. (2013). Differential expression of three members of the multidomain adhesion CCp family in *Babesia bigemina*, *Babesia bovis* and *Theileria equi*. PLoS ONE.

[36] Alzan H.F., Bastos R.G., Ueti M.W., Laughery J.M., Rathinasamy V.A., Cooke B.M., Suarez C.E. (2021). Assessment of *Babesia bovis* 6cys A and 6cys B as components of transmission blocking vaccines for babesiosis. Parasites Vectors.

[37] Alzan H.F., Cooke B.M., Suarez C.E. (2019). Transgenic *Babesia bovis* lacking 6-Cys sexual-stage genes as the foundation for non-transmissible live vaccines against bovine babesiosis. Ticks Tick Borne Dis..

[38] Alzan H.F., Lau A.O., Knowles D.P., Herndon D.R., Ueti M.W., Scoles G.A., Kappmeyer L.S., Suarez C.E. (2016). Expression of 6-Cys gene superfamily defines *Babesia bovis* sexual stage development within *Rhipicephalus microplus*. PLoS ONE.

[39] Li J., Zheng J., Gong P., Zhang X. (2012). Efficacy of *Eimeria tenella* rhomboid-like protein as a subunit vaccine in protective immunity against homologous challenge. Parasitol. Res..

[40] Zhang N.Z., Xu Y., Wang M., Petersen E., Chen J., Huang S.Y., Zhu X.Q. (2015). Protective efficacy of two novel DNA vaccines expressing *Toxoplasma gondii* rhomboid 4 and rhomboid 5 proteins against acute and chronic toxoplasmosis in mice. Expert Rev. Vaccines.

[41] Han Y., Zhou A., Lu G., Zhao G., Wang L., Guo J., Song P., Zhou J., Zhou H., Cong H. (2017). Protection via a ROM4 DNA vaccine and peptide against *Toxoplasma gondii* in BALB/c mice. BMC Infect. Dis..

[42] Urban S. (2009). Making the cut: Central roles of intramembrane proteolysis in pathogenic microorganisms. Nat. Rev. Microbiol..

[43] Strisovsky K. (2016). Rhomboid protease inhibitors: Emerging tools and future therapeutics. Semin. Cell Dev. Biol..

[44] Tichá A., Stanchev S., Vinothkumar K.R., Mikles D.C., Pachl P., Began J., Škerle J., Švehlová K., Nguyen M.T.N., Verhelst S.H.L. (2017). General and modular strategy for designing potent, selective, and pharmacologically compliant inhibitors of rhomboid proteases. Cell Chem. Biol..

[45] Gandhi S., Baker R.P., Cho S., Stanchev S., Strisovsky K., Urban S. (2020). Designed parasite-selective rhomboid inhibitors block invasion and clear blood-stage malaria. Cell Chem. Biol..

[46] Brayton K.A., Lau A.O., Herndon D.R., Hannick L., Kappmeyer L.S., Berens S.J., Bidwell S.L., Brown W.C., Crabtree J., Fadrosh D. (2007). Genome sequence of *Babesia bovis* and comparative analysis of apicomplexan hemoprotozoa. PLoS Pathog..

[47] Bustin S.A., Beaulieu J.F., Huggett J., Jaggi R., Kibenge F.S., Olsvik P.A., Penning L.C., Toegel S. (2010). MIQE précis: Practical implementation of minimum standard guidelines for fluorescence-based quantitative real-time PCR experiments. BMC Mol. Biol..

[48] Ramakers C., Ruijter J.M., Deprez R.H., Moorman A.F. (2003). Assumption-free analysis of quantitative real-time polymerase chain reaction (PCR) data. Neurosci. Lett..

[49] Pfaffl M.W., Horgan G.W., Dempfle L. (2002). Relative expression software tool (REST) for group-wise comparison and statistical analysis of relative expression results in real-time PCR. Nucleic Acids Res..

[50] Di Rienzo J.A. (2009). fgStatistics. Statistical Software for the Analysis of Experiments of Functional Genomics.

[51] Hines S.A., Palmer G.H., Jasmer D.P., McGuire T.C., McElwain T.F. (1992). Neutralization-sensitive merozoite surface antigens of *Babesia bovis* encoded by members of a polymorphic gene family. Mol. Biochem. Parasitol..

[52] Rodriguez S.D., Buening G.M., Green T.J., Carson C.A. (1983). Cloning of *Babesia bovis* by in vitro cultivation. Infect. Immun..

[53] Anziani O.S., Guglielmone A.A., Abdala A.A., Aguirre D.H., Mangold A.J. (1993). Protección conferida por *Babesia bovis* vacunal en novillos Holando Argentino. Rev. Med. Vet..

[54] Flores D.A., Minichiello Y., Araujo F.R., Shkap V., Benítez D., Echaide I., Rolls P., Mosqueda J., Pacheco G.M., Petterson M. (2013). Evidence for extensive genetic diversity and substructuring of the *Babesia bovis* metapopulation. Transbound. Emerg. Dis..

[55] Echaide I.E., de Echaide S.T., Mangold A.J., Guglielmone A.A. Live and soluble antigens from in vitro culture to vaccinate cattle against *Babesia bovis*. Proceedings of the IX International Veterinary Hemoparasite Disease Conference.

[56] Ramos C.A.N., Araujo F.R., Alves L.C., Fernando de Souza I.I., Guedes D.S., Soares C.O. (2012). Genetic conservation of potentially immunogenic proteins among Brazilian isolates of *Babesia bovis*. Vet. Parasitol..

[57] Mazuz M.L., Molad T., Fish L., Leibovitz B., Wolkomirsky R., Fleiderovitz L., Shkap V. (2012). 2012: Genetic diversity of *Babesia bovis* in virulent and attenuated strains. Parasitology.

[58] Bock R.E., Blight G.W., Kingston T.G., de Vos A.J. (1995). A survey of cattle producers in the *Boophilus microplus* endemic area of Queensland to determine attitudes to the control of and vaccination against tick fever. Aust. Vet. J..

