# Supplementary material for: Unraveling the Complexity of the Rhomboid Serine Protease 4 Family of Babesia bovis Using Bioinformatics and Experimental Studies
Source: Pathogens. 2022 Mar 12;11(3):344. doi: 10.3390/pathogens11030344 (PMC8956091; doi:10.3390/pathogens11030344)
Supplement: Supplementary file 1 [file pathogens-11-00344-s001.zip › pathogens-1577753-supplementary.pdf]

Supplementary Figure S1. Clustal omega alignment of ROM4 paralogs of *B. bovis*, T2Bo strain

```
4_1      ------------------------------------------------------------       0
4_2      ------------------------------------------------------------       0
4_5      MGDNDSNDKKNANRHSLNAIKGGFVKDMVNRGFRKKINDSDKNDDSKEDKGGEKPSNVSK       60
4_3      MGDNDSNDKKNANRHSLNAIKGGFVKDMVNRGFRKKINDSDKNDDSNVDGPSNDIT----       56
4_4      MGDNDSNDKKNANRHSLNAIKGGFVKDMVNRGFRKKINDSDKNDDSNVDGPSNDIT----       56


4_1      ------------------------------------------------------------       0
4_2      ------------------------------------------------------------       0
4_5      DSSSMKAGNVAALRDQFKRAAEKDESKDQNSQAGQSSLTSKAPLGDPRTDQRARAKFLAE       120
4_3      DKGHHRNA-------------GIEDSENILSQA-----------QSDTKDKPSVAKSLAE       92
4_4      DKGHHRNA-------------GIEDSENILSQA-----------QSDTKDKPSVAKSLAE       92


4_1      ------------------------------------------------------------       0
4_2      ------------------------------------------------------------       0
4_5      KYEKILAARNMVEESASRALQRPATSTPAKIPASVSGVSKEPKDTSEQPRPSKAEPRPST       180
4_3      KYERGLFNRNKVDE----------------------------------------------       106
4_4      KYERGLFNRNKVDE----------------------------------------------       106


4_1      ------------------------------------------------------------       0
4_2      ------------------------------------------------------MPCKYP       6
4_5      TPSTSLGPQEPKDTSEQPRPSKAEPRPSTTPSTSLGPQEPKDTSEQPRPSKAEPRPSTTP       240
4_3      ---------------------------------------------SLISKAESQPSTTP       120
4_4      ---------------------------------------------SLISKAESQPSTTP       120


4_1      -------------------------MKDDEIEFS-------------------------       9
4_2      SV-----------------------------------------------SGVS--IDR       15
4_5      STSLGPQEPKDTSEQPRSSKAESKLSTTLSKLRFSRESRSVIKGTGSFGGSSQVSSNAGR       300
4_3      STSMGS---------------------------------SEIKGARAFGGSSQVSSNAGR       147
4_4      STSMGS---------------------------------SEIKGARAFGGSSQVSSNAGR       147


4_1      -----DEEEPTQTGEVHLA---SPEIIEAPQKVPKTKVVINRRSPINPLE--KLAYVAGF       59
4_2      WESALYDEKPVKTVEITQCSTAEPEEVSTTGA----------EPPSEAMTHRLCKWIFDL       65
4_5      RVHTLADTASTDDASIRQA---MSQSFSSPGV----------SQPEQPERASRGRNMFGM       347
4_3      RVHTLADTASTDDASIRQA---MSQSFSSPGV----------SQPEQPERASRGRNMFGM       194
4_4      RVHTLADTASTDDASIRQA---MSQSFSSPGV----------SQPEQPERASRGRNMFGM       194
               :   ..  .:  .     : ..:               * :         : .:

4_1      KNNEDNNIVKGSDPMLEKNPLLGRLPFMLMINFALVLIFITQLVFNYITFNGRCISSVLY       119
4_2      VNKLKYCIRK----DSADHDVSGKLIVLRTALIIYSTAYICSLVLNKTTFDGKCMGNVDF       121
4_5      RR-RPCWIRI----QVKEKIFPGKMILAISTTILIIVVFMFELFINRATFNGRCIGEVDY       402
4_3      RR-RPCWIRI----QVKEKIFPGKMVLAISTTILMIVVFLFELLLNKTTFNGRCIGQVDY       249
4_4      RR-RPCWIRI----QVKEKIFPGKMVLAISTTILMIVVFLFELLLNKTTFNGRCIGQVDY       249
          .     *         .: . *:: .     :     :: .*.:*  **:*:*:..* :

4_1      PAIQTGREAIKPYIVEFGYGACEYNLKTSTANRVFFGESASDEGWPKEYLQKLVTAASSA       179
4_2      GYGTLG----HPFHSPLGYNACTQNTKTKIIEPNFHIHQNTEGQQVLVWKENERWH---D       174
4_5      THRHEG----KAILSYLGYVGCENNLKTTAAERGFLGAGASDKGYPVDYVHKGNLATSLA       458
4_3      SNRLAG----KSTLSFIGYVGCENNLKTTAAERGFLGAAASDAGYPDDIVTDGRLAHAPA       305
4_4      SNRLAG----KSTLSFIGYVGCENNLKTTAAERGFLGAAASDAGYPDDIVTDGRLAHAPA       305
              *    :     :** .*  * **.  :  *     ::         .

4_1      SSDAPNERVYNLLGGLSTNVMRNYEEYYRVFWSMFLHSSWKHLFFNVFCQIQALWIIEPD       239
4_2      RISEPGSQSLTVLGAVDTNHIRIYGEWFRIFTGIILHAGWFHLLNNALIHFIVLYIIEPE       234
4_5      PPDGPNYRIGTIVGALSANTIRIYGESSRLLTSIFLHGGRWHLLCNCLMNMLLLYVIEPD       518
4_3      VVDGPNVRIASIIGAVSANEVRLYNEKFRILSAIFLHAGFTHLFTNCLMNMLLLYVIEPD       365
4_4      VVDGPNVRIASIIGAVSANEVRLYNEKFRILSAIFLHAGFTHLFTNCLMNMLLLYVIEPD       365
           . *. :  .::*.:.:* :* * *  *:: .::**..  **: * : ::  *::***:

4_1      WGFMRTMGLFLISGIGGNLTGAVLSPCGLTVGSSGAMYGLYGAMIPYCIEYWNTIPRPVF       299
4_2      WGFYRTLLGYFVTGCGSYLAGAVFIPCLRQIGSSGVHFGFIGAIAPYCVENWYRMGSPVI       294
4_5      WGFKRTLALYIFGGYSANLVHASMSPCIPCWGASGSLFSLYGAFIPYTVEHWDNLRSPMA       578
4_3      WGFKRTSLLYLVAGIGGNLVHTSMSPCIPCWGASGSLFGLYGAFIPYTIEHWDNLRSPLL       425
4_4      WGFKRTSLLYLVAGIGGNLVHTSMSPCIPCWGASGSLFGLYGAFIPYTIEHWDNLRSPLL       425
         *** **   ::. * .. *. : : **    *:**  :.: **: ** :* *  :  *:

4_1      LFCYNIITLIIGFLMGLAPNVDNYCHIGGCVFGMLWGFATIKSVSSCDKCTIVERSLLCP       359
4_2      VLSLSLLVPLLDLCI-KEADIGIQIHLGGYIFGMLYGFSTIKAVTLFDKGVPYYRFTIKF       353
4_5      LIVIAITISLLEIIL-PGVGVSNHAHLGGFAFGLCFGFATLKSVSAFDRGALWSRIALRF       637
4_3      LLLISVAFVSIEFFS-FVRGVSKHAHLGGFAFGLCFGFATLKSVSAFDRGALWSRIALRF       484
4_4      LLLISVAFVSIEFFS-FVRGVSKHAHLGGFAFGLCFGFATLKSVSAFDRGALWSRIALRF       484
         ::   :    : :      .:.   *:**  **: :**:*:*:*:  *: .   *  :

4_1      LISWALPQKWKAKLRLIIVFKKDRGDRNRQKDMAQKHAVQNGVTPRRIALIKKKFERQGA       419
4_2      FSRWLKESTRN--LYIRKVVKACQIEE----MNRIQYENQAKSSTCGFFFMKRLFGVYPL       407
4_5      SSRLSPEAVQK---HQAKVVKSAQSEE----ILRIKYEQGAKDSANRLRFVKRMFGIYPY       690
4_3      SSRLSPEAVQK---HQAKVVKSAQSEE----ILRIKYEQGAKDSANRLRFVKRMFGIYPY       537
4_4      SSRLSPEAVQK---HQAKVVKSAQSEE----ILRIKYEQGAKDSANRLRFVKRMFGIYPY       537
                   :       *.*  : :.        ::      :   : ::*: *

4_1      PPCRMRLREWIFRITSLLMMVL------------LFTILGLFLSKPELYAKFKPPGEYKL       467
4_2      GPYRMRPRDWVTRGISFCLMVI------------LMTFLILMLCFEPLYSSFNPTVSSAF       455
4_5      GPYRMRLRDVLTRVVFLCAL------------IAMFVFFYLATFYESVYKNIDRNATILF       738
4_3      GPYRMRLRDVLTRVVFLCAL------------IAMFVFFYYSTFNERFYTWLNRAGIRFV       585
4_4      GPYRMRLRDVLTRVVFLCALVSQPVFTYTPEQIAMFVFFYYSTFNERFYTWLNRAGIRFV       597
          * *** *: : *   :  :              ::.::        .*  :.      .

4_1      SGWQTCECCFITNIEKLFSGHRDIPAQYL-NRKLFWCFNNVDDAKHFCGDDYAKTAPMDN       526
4_2      --TQTCICCYIKIRNKSAL----QRLQVLDLAGKYFCFRSKEYANRYCF----------       498
4_5      --SRACHCGYFKS-SGDEL----LLNQIGNLAGKFYCFDSESARDKFCAVSG--------       783
4_3      --SPLCHCGYLRDLTGPSI----LSKQIGSLAGKYYCFQSEALWNVFCEK----------       629
4_4      --SPLCHCGYLRDLTGPSI----LSKQIGSLAGKYYCFQSEALWNVFCEK----------       641
              * * ::               *       ::** .    . :*

4_1      ILKSSHDAALGLINTVTEAIK        547
```

```
4_2      ---------------------        498
4_5      ---------------------        783
4_3      ---------------------        629
4_4      ---------------------        641
```

Supplementary Figure S2. Clustal omega alignment of intergenic regions upstream to *rom4.3* (Int3), *rom4.4* (Int4) and *rom4.5* (Int5)

```
Int3      TTTGGTGCACAGACTAGACTATCAGAATAATGGCCACGAGCACATCTCACACATATGTAA      60
Int5      TTTGGTGCACAGACTAGACTATCAGAATAATGGCCACGAGCACATCTCACACATATGTAA      60
Int4      TTTGGTACACAAACTATACTATCACAATAATGGCCACGAGCACATCTCACACATATGTAA      60
          ****** **** **** ******* ***********************************

Int3      ACAAAAAACCAACACACGCCAAAAATGGCAAATCTAGTATATTAGATGATAAAAGGTCGT      120
Int4      ACAAAAAACCAACACACGCGGGAAATAGTAATATTTTTATAGTAGTCTATAAAGAAATGT      120
Int5      ACAAAAAACCAACACACGCGGGAAATAGTAATGTTTTTATATTAAATTATAAAGAGATGT      120
          *******************   **** * **   *  **** **    *****     **

Int3      TATTTTAAAGAATCATTACACAGAGCACTGCACCGTCATAGGAATAAATAACGTCGTGCA      180
Int4      GATGTCGATATATTCGTGAGTTG-CATATCTCGCACATCAACGTGAATAATCAATAATAA      179
Int5      AATATTAAAGAATCTTCTTTAAA-GTACTGCACCGTCACATGAATAAATAACCCGGTGCA      179
           ** *  *   **               *    *     *     **  * *       *

Int3      TGCAAGTCTTCCTAAAAAGAATAACTAAAATAATAAATGACGAAGCCATTTAGTGAAACA      240
Int4      CGCATGTAATTAGGGATGCAACAC--ACGCCCAATTACACATCAAGGGGTAAATAGAACA      237
Int5      TGCACGTCCTTCAGATAAGAATAA--GTAAAAATGAATATTACGGCTAATCAATAGAACA      237
           *** **  *         ** *         *   *            * * *  ****

Int3      CATCCATTGATGACACATGTTTTCGTGTTGTATATTAGTTTTACGTGTATACACCCTGGG      300
Int4      CATCCATTGATGACACATGTTTTCGTGTTGTATATTAGTTTTACGTGTATACACCCTGGG      297
Int5      CATCCATTGATGACATATGTTTTCGTGTTGGATATTAGTTTTACGTGTATACAGCCTAGG      297
          *************** ************** ********************** *** **

Int3      TAATATATATACAATCCCATCACACATCTGTCTGTATACAAAACGAATATCAGTCGTCTA      360
Int4      TAATATATATACAATCCCATCACACAGCTGCCTATATAAA---ATAATATAAGTCGCCTA      354
Int5      TAATATATCTACAATCCCATCACACAGCTGCCTATATAAA---ATAATATAAGTCGCCTA      354
          ******** ***************** *** ** **** *     ***** ***** ***

Int3      CACACTTTGTAACAAACCGAATGTGTAGTGCATTAGACCGGTAAACACAATGCTAATAGA      420
Int4      CACACTTTGTAACAAACCGAATGTGTAGTGCATTAGACCGGTAAACACAATGCTAATAGA      414
Int5      CACACTTTGTAACAAACCGAATGTGTAGTGCATTAGACCGGTAAACACAATGCTAATAGA      414
          ************************************************************

Int3      GGCATAAAACCACAAAGGGTCACTGAATAAAACGGTAGATTACATAT-------------      467
Int4      GGCATAAAACCACAAAGGGTCACTGAATAAAACGGTAGATTACATATAACGTGTTATATG      474
Int5      GGCATAAAACCACAAAGGGTCACTGAATAAAACGGTAGATTACATATAACGTGTTATATG      474
          ***********************************************

Int3      ------------------------------------------------------------      467
Int4      CCTTGTGGTCAACAACTTTACCACGTTTAGTGATTGGCGCCATTCCCGTCAATAGTACCT      534
Int5      CCTTGTGGTCAACAACTTTACCACGTTTAGTGATTGGCGCCATTCCCGTCAATAGTACCT      534


Int3      ------------------------------------------------------------      467
Int4      TATTTACGACGATATAGCACAGATAATGGTCTAACAAAAGTATGTTACACATTGTTTCAC      594
Int5      TATTTACGACGATATAGCACAGATAATGGTCTAACAAAAGTATGTTACACATTGTTTCAC      594


Int3      ------------------------------------------------------------      467
Int4      ATGCTTACGCAAGGAAGATGCGCAGTCGGTTTCGACCGCAGGGCAGAAGTGGTTCAACCA      654
Int5      ATGCTTACGCAAGGAAGATGCGCAGTCGGTTTCGACCGCAGGGCAGAAGTGGTTCAACCA      654


Int3      ------------------------------------------------------------      467
Int4      GTCTAACAGCATTTATCTTTTCTAATCGCTGATTGACTTTTTACATAGTGATGAATGTGA      714
Int5      GTCTAACAGCATTTATCTTTTCTAATCGCTGATTGACTTTTTACATAGTGATGAATGTGA      714


Int3      ---------------------------------        467
Int4      TAAGTCACCTATGGTTAATACCACTGCTTGCTA        747
Int5      TAAGTCACCTATGGTTAATACCACTGCTTGCTA        747
```

Supplementary Figure S3. Melting curve analysis to select an adequate housekeeping gene as normalization control of transcription in *B. bovis*, as determined by qPCR. Dissociation curves were obtained when primer pairs targeting topoisomerase (topo 1: a, e; topo 2: b, f), gadph (c, g) or actin (d, h) and plasmid (a-d) or cDNA from AS and SS (e-h), and StepOne Software v2.3 were employed.

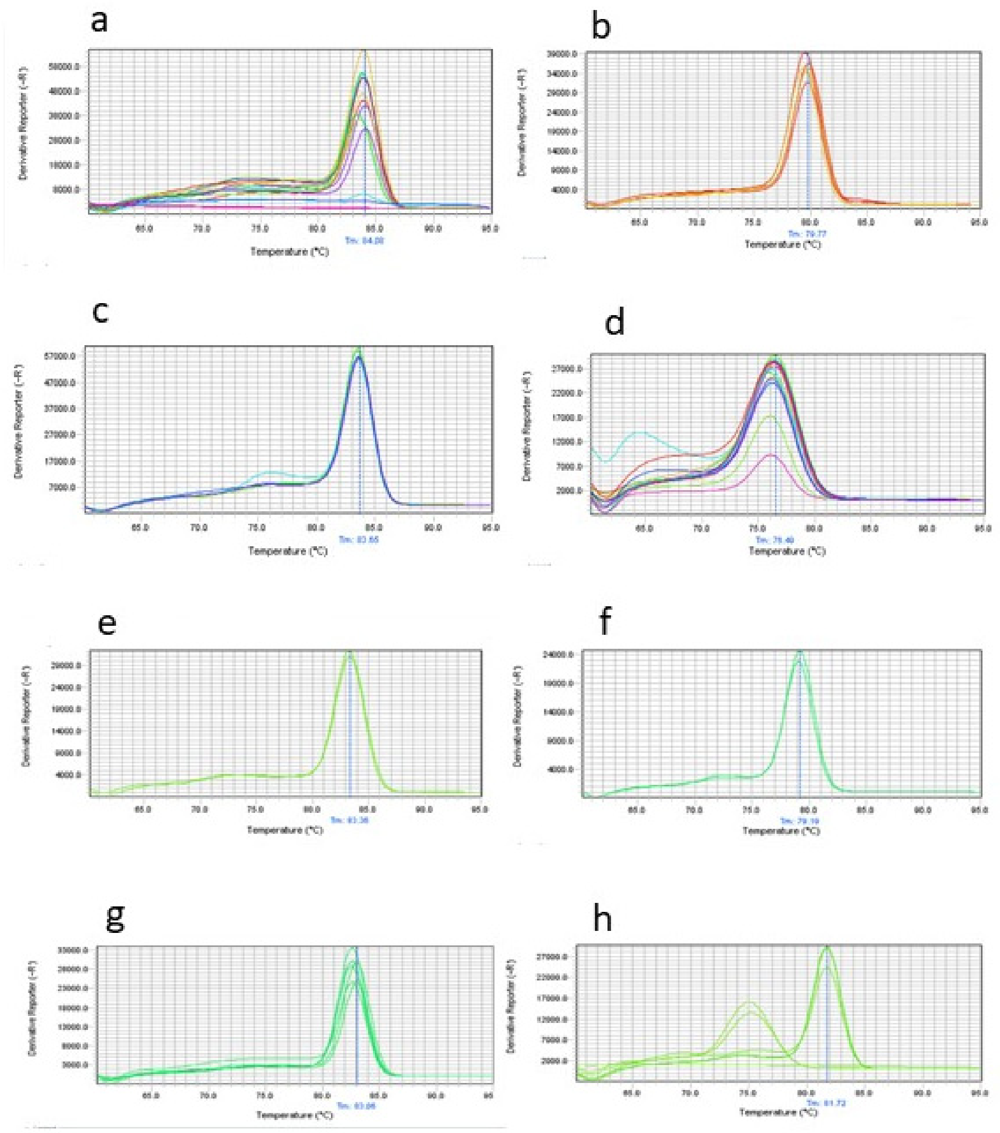

Supplementary Figure S4. Clustal omega alignment of nucleotide sequences of *rom4.5* alleles of *Babesia bovis* T2B and R1A. Note total conservation along the sequences with the exception of a region of different length which encodes the 29-aa repeats.

```
R1A      ATGGGAGATAATGATTCGAATGATAAGAAGAATGCTAATAGGCATTCACTTAACGCTATT      60
T2B      ATGGGAGATAATGATTCGAATGATAAGAAGAATGCTAATAGGCATTCACTTAACGCTATT      60
         ************************************************************

R1A      AAAGGCGGTTTTGTGAAGGATATGGTGAATCGGGGATTCAGGAAGAAGATTAATGATAGT      120
T2B      AAAGGCGGTTTTGTGAAGGATATGGTGAATCGGGGATTCAGGAAGAAGATTAATGATAGT      120
         ************************************************************

R1A      GATAAGAATGATGATAGCAAAGAGGATAAAGGTGGAGAAAAACCGAGTAACGTGTCGAAG      180
T2B      GATAAGAATGATGATAGCAAAGAGGATAAAGGTGGAGAAAAACCGAGTAACGTGTCGAAG      180
         ************************************************************

R1A      GATTCGTCGTCTATGAAAGCTGGTAATGTGGCTGCTTTGAGAGATCAATTCAAGCGTGCT      240
T2B      GATTCGTCGTCTATGAAAGCTGGTAATGTGGCTGCTTTGAGAGATCAATTCAAGCGTGCT      240
         ************************************************************

R1A      GCTGAGAAAGATGAATCTAAAGACCAAAATTCGCAAGCTGGGCAGAGCAGCTTAACCTCG      300
T2B      GCTGAGAAAGATGAATCTAAAGACCAAAATTCGCAAGCTGGGCAGAGCAGCTTAACCTCG      300
         ************************************************************

R1A      AAGGCTCCTCTTGGTGATCCGAGAACTGATCAACGAGCACGAGCCAAATTCCTCGCAGAG      360
T2Bo     AAGGCTCCTCTTGGTGATCCGAGAACTGATCAACGAGCACGAGCCAAATTCCTCGCAGAG      360
         ************************************************************

R1A      AAATATGAAAAAATATTGGCTGCCCGTAACATGGTGGAAGAGTCCGCCAGTCGTGCATTG      420
T2Bo     AAATATGAAAAAATATTGGCTGCCCGTAACATGGTGGAAGAGTCCGCCAGTCGTGCATTG      420
         ************************************************************

R1A      CAGCGCCCAGCGACGAGTACACCAGCAAAGATACCGGCCTCTGTATCTGGTGTTTCGAAA      480
T2B      CAGCGCCCAGCGACGAGTACACCAGCAAAGATACCGGCCTCTGTATCTGGTGTTTCGAAA      480
         ************************************************************

R1A      GAACCTAAAGACACCAGCGAACAACCTAGGCCATCGAAAGCTGAACCTCGACCTAGTACC      540
T2B      GAACCTAAAGACACCAGCGAACAACCTAGGCCATCGAAAGCTGAACCTCGACCTAGTACC      540
         ************************************************************

R1A      ACACCATCTACCTCCTTAGGTCCACAAGAACCTAAAGACACCAGCGAACAACCTAGGCCA      600
T2B      ACACCATCTACCTCCTTAGGTCCACAAGAACCTAAAGACACCAGCGAACAACCTAGGCCA      600
         ************************************************************

R1A      TCGAAAGCTGAACCTCGACCTAGTACCACACCATCTACCTCCTTAGGTCCACAAGAACCT      660
T2B      TCGAAAGCTGAACCTCGACCTAGTACC---------------------------------      627
         ***************************

R1A      AAAGACACCAGCGAACAACCTAGGCCATCGAAAGCTGAACCTCGACCTAGTACCACACCA      720
T2B      ------------------------------------------------------------      627


R1A      TCTACCTCCTTAGGTCCACAAGAACCTAAAGACACCAGCGAACAACCTAGGCCATCGAAA      780
T2B      ------------------------------------------------------------      627


R1A      GCTGAACCTCGACCTAGTACCACACCATCTACCTCCTTAGGTCCACAAGAACCTAAAGAC      840
T2B      ------------------------------------------------------------      627


R1A      ACCAGCGAACAACCTAGGCCATCGAAAGCTGAACCTCGACCTAGTACCACACCATCTACC      900
T2B      ------------------------------------------------------------      627


R1A      TCCTTAGGTCCACAAGAACCTAAAGACACCAGCGAACAACCTAGGCCATCGAAAGCTGAA      960
T2B      ------------------------------------------------------------      627


R1A      CCTCGACCTAGTACCACACCATCTACCTCCTTAGGTCCACAAGAACCTAAAGACACCAGC      1020
T2B      ------------------------------------------------------------      627


R1A      GAACAACCTAGGCCATCGAAAGCTGAACCTCGACCTAGTACCACACCATCTACCTCCTTA      1080
T2B      ------------------------------------------ACACCATCTACCTCCTTA      645
                                                   ******************

R1A      GGTCCACAAGAACCTAAAGACACCAGCGAACAACCTAGGCCATCGAAAGCTGAACCTCGA      1140
T2B      GGTCCACAAGAACCTAAAGACACCAGCGAACAACCTAGGCCATCGAAAGCTGAACCTCGA      705
         ************************************************************

R1A      CCTAGTACCACACCATCTACCTCCTTAGGTCCACAAGAACCTAAAGACACCAGCGAACAA      1200
T2B      CCTAGTACCACACCATCTACCTCCTTAGGTCCACAAGAACCTAAAGACACCAGCGAACAA      765
         ************************************************************

R1A      CCCAGATCATCGAAAGCTGAGTCCAAATTGAGTACTACACTATCAAAATTGAGATTCTCT      1260
T2B      CCCAGATCATCGAAAGCTGAGTCCAAATTGAGTACTACACTATCAAAATTGAGATTCTCT      825
         ************************************************************

R1A      CGTGAATCCCGATCAGTGATTAAAGGTACGGGCTCTTTTGGCGGTTCATCTCAGGTAAGC      1320
T2Bo     CGTGAATCCCGATCAGTGATTAAAGGTACGGGCTCTTTTGGCGGTTCATCTCAGGTAAGC      885
         ************************************************************

R1A      TCCAATGCTGGTCGTCGTGTTCATACTTTGGCGGACACTGCTTCCACTGATGACGCTAGT      1380
T2B      TCCAATGCTGGTCGTCGTGTTCATACTTTGGCGGACACTGCTTCCACTGATGACGCTAGT      945
         ************************************************************

R1A      ATTAGACAGGCTATGTCCCAATCCTTTTCCAGCCCTGGTGTTAGTCAGCCAGAGCAACCC      1440
T2B      ATTAGACAGGCTATGTCCCAATCCTTTTCCAGCCCTGGTGTTAGTCAGCCAGAGCAACCC      1005
         ************************************************************
```

```
R1A       GAGCGTGCCTCGCGTGGCCGTAACATGTTTGGTATGCGTCGCCGTCCATGTTGGATTCGT      1500
T2B       GAGCGTGCCTCGCGTGGCCGTAACATGTTTGGTATGCGTCGCCGTCCATGTTGGATTCGT      1065
          ************************************************************

R1A       ATTCAAGTGAAGGAGAAGATATTTCCCGGTAAGATGATATTGGCAATCAGTACTACAATA      1560
T2B       ATTCAAGTGAAGGAGAAGATATTTCCCGGTAAGATGATATTGGCAATCAGTACTACAATA      1125
          ************************************************************

R1A       CTGATAATTGTTGTTTTTATGTTTGAGTTATTCATCAACAGAGCTACCTTTAACGGTCGC      1620
T2B       CTGATAATTGTTGTTTTTATGTTTGAGTTATTCATCAACAGAGCTACCTTTAACGGTCGC      1185
          ************************************************************

R1A       TGTATAGGTGAGGTGGATTATACTCACAGACATGAGGGTAAAGCTATCTTATCATACCTG      1680
T2B       TGTATAGGTGAGGTGGATTATACTCACAGACATGAGGGTAAAGCTATCTTATCATACCTG      1245
          ************************************************************

R1A       GGTTATGTTGGTTGTGAGAATAACCTCAAAACAACTGCTGCCGAACGTGGATTCCTTGGT      1740
T2B       GGTTATGTTGGTTGTGAGAATAACCTCAAAACAACTGCTGCCGAACGTGGATTCCTTGGT      1305
          ************************************************************

R1A       GCTGGAGCGAGTGATAAAGGCTATCCAGTTGATTATGTGCATAAAGGGAATTTGGCAACT      1800
T2B       GCTGGAGCGAGTGATAAAGGCTATCCAGTTGATTATGTGCATAAAGGGAATTTGGCAACT      1365
          ************************************************************

R1A       TCACTTGCTCCTCCTGACGGACCTAATTACCGTATAGGCACTATTGTTGGCGCACTATCG      1860
T2Bo      TCACTTGCTCCTCCTGACGGACCTAATTACCGTATAGGCACTATTGTTGGCGCACTATCG      1425
          ************************************************************

R1A       GCTAATACAATTCGTATCTATGGAGAGTCCTCTAGGTTGCTTACTTCCATATTCTTACAT      1920
T2B       GCTAATACAATTCGTATCTATGGAGAGTCCTCTAGGTTGCTTACTTCCATATTCTTACAT      1485
          ************************************************************

R1A       GGAGGAAGGTGGCATCTTCTTTGTAATTGTTTGATGAACATGTTGCTTCTCTATGTGATT      1980
T2B       GGAGGAAGGTGGCATCTTCTTTGTAATTGTTTGATGAACATGTTGCTTCTCTATGTGATT      1545
          ************************************************************

R1A       GAGCCGGTAAGTGGCCACTTTTGTTTTGCTAATCATATTCTAGGACTGGGGATTCAAACG      2040
T2B       GAGCCGGTAAGTGGCCACTTTTGTTTTGCTAATCATATTCTAGGACTGGGGATTCAAACG      1605
          ************************************************************

R1A       CACATTAGCGCTGTATATATTCGGTGGTTACAGTGCCAATCTGGTTCACGCCAGTATGTC      2100
T2B       CACATTAGCGCTGTATATATTCGGTGGTTACAGTGCCAATCTGGTTCACGCCAGTATGTC      1665
          ************************************************************

R1A       TCCTTGTATCCCATGCTGGGGAGCTTCTGGGTCACTGTTTAGTTTGTATGGAGCATTCAT      2160
T2B       TCCTTGTATCCCATGCTGGGGAGCTTCTGGGTCACTGTTTAGTTTGTATGGAGCATTCAT      1725
          ************************************************************

R1A       TCCTTACACGGTAGAGCATTGGGACAACCTGAGATCACCAATGGCTCTAATTGTCATAGC      2220
T2B       TCCTTACACGGTAGAGCATTGGGACAACCTGAGATCACCAATGGCTCTAATTGTCATAGC      1785
          ************************************************************

R1A       CATAACAATCTCTCTCCTGGAAATAATATTACCGGGAGTAGGCGTTAGTAATCATGCCCA      2280
T2B       CATAACAATCTCTCTCCTGGAAATAATATTACCGGGAGTAGGCGTTAGTAATCATGCCCA      1845
          ************************************************************

R1A       CTTGGTAGGTTTTTATTTGCTATGTTAATGAATACACAGGGTGGTTTTGCGTTTGGTTTG      2340
T2B       CTTGGTAGGTTTTTATTTGCTATGTTAATGAATACACAGGGTGGTTTTGCGTTTGGTTTG      1905
          ************************************************************

R1A       TGTTTCGGTTTCGCCACATTGAAATCAGTATCTGCGTTTGACCGTGGTGCCTTGTGGTCA      2400
T2B       TGTTTCGGTTTCGCCACATTGAAATCAGTATCTGCGTTTGACCGTGGTGCCTTGTGGTCA      1965
          ************************************************************

R1A       CGTATAGCACTTCGGTTTTCTAGTAGGCTTTCACCAGAGGCCGTGCAGAAACACCAAGCC      2460
T2B       CGTATAGCACTTCGGTTTTCTAGTAGGCTTTCACCAGAGGCCGTGCAGAAACACCAAGCC      2025
          ************************************************************

R1A       AAGGTTGTGAAGAGTGCTCAGTCAGAGGAGATTCTGCGTATTAAATATGAGCAGGGTGCT      2520
T2B       AAGGTTGTGAAGAGTGCTCAGTCAGAGGAGATTCTGCGTATTAAATATGAGCAGGGTGCT      2085
          ************************************************************

R1A       AAGGATAGTGCTAATCGTCTTCGCTTTGTCAAGCGTATGTTCGGTATATATCCCTATGGC      2580
T2B       AAGGATAGTGCTAATCGTCTTCGCTTTGTCAAGCGTATGTTCGGTATATATCCCTATGGC      2145
          ************************************************************

R1A       CCATACCGTATGAGGCTGCGTGATGTACTTACTCGCGTTGTATTCCTTTGTGCCCTAGTA      2640
T2B       CCATACCGTATGAGGCTGCGTGATGTACTTACTCGCGTTGTATTCCTTTGTGCCCTAGTA      2205
          ************************************************************

R1A       AGTCAACCGGTATTTACATACACTCCTGAGCAGATCGCGATGTTTGTATTCTTCTATTTG      2700
T2B       AGTCAACCGGTATTTACATACACTCCTGAGCAGATCGCGATGTTTGTATTCTTCTATTTG      2265
          ************************************************************

R1A       GCGACTTTCTATGAAAGCGTCTATAAGAATATAGATAGGAATGCAACGATACTTTTCTCT      2760
T2B       GCGACTTTCTATGAAAGCGTCTATAAGAATATAGATAGGAATGCAACGATACTTTTCTCT      2325
          ************************************************************

R1A       CGTGCCTGCCATTGCGGCTATTTCAAATCATCTGGTGATGAGTTGTTATTAAATCAAATA      2820
T2B       CGTGCCTGCCATTGCGGCTATTTCAAATCATCTGGTGATGAGTTGTTATTAAATCAAATA      2385
          ************************************************************

R1A       GGCAACCTAGCTGGCAAATTCTATTGTTTCGATTCAGAAAGCGCCAGGGATAAGTTCTGT      2880
T2B       GGCAACCTAGCTGGCAAATTCTATTGTTTCGATTCAGAAAGCGCCAGGGATAAGTTCTGT      2445
          ************************************************************

R1A       GCTGTTTCTGGTTGAACCGGTTAACATTGTATCACGTACATGCTGAACTTTTCATCATT       2939
T2B       GCTGTTTCTGGTTGAACCGGTTAACATTGTATCACGTACATGCTGAACTTTTCATCATT       2504
          ***********************************************************
```

Supplementary Figure S5. Clustal omega alignment of the N-terminal region of ROM4.5 of *Babesia bovis* T2Bo and R1A. Repeats in the R1A sequence are highlighted with different colors and the aa in the conserved motifs that flank the repeats' region are shown in red.

```
Rom4.1_R1A      MGDNDSNDKKNANRHSLNAIKGGFVKDMVNRGFRKKINDSDKNDDSKEDKGGEKPSNVSK 60
Rom4.1_T2B      MGDNDSNDKKNANRHSLNAIKGGFVKDMVNRGFRKKINDSDKNDDSKEDKGGEKPSNVSK 60
                ************************************************************

Rom4.1_R1A      DSSSMKAGNVAALRDQFKRAAEKDESKDQNSQAGQSSLTSKAPLGDPRTDQRARAKFLAE 120
Rom4.1_T2B      DSSSMKAGNVAALRDQFKRAAEKDESKDQNSQAGQSSLTSKAPLGDPRTDQRARAKFLAE 120
                ************************************************************

Rom4.1_R1A      KYEKILAARNMVEESASRALQRPATSTPAKIPASVSGVSKEPKDTSEQPRPSKAEPRPST 180
Rom4.1_T2B      KYEKILAARNMVEESASRALQRPATSTPAKIPASVSGVSKEPKDTSEQPRPSKAEPRPST 180
                ************************************************************

Rom4.1_R1A      TPSTSLGPQEPKDTSEQPRPSKAEPRPSTTPSTSLGPQEPKDTSEQPRPSKAEPRPSTTP 240
Rom4.1_T2B      TPSTSLGPQEPKDTSEQPRPSKAEPRPSTTP----------------------------- 211
                *******************************

Rom4.1_R1A      STSLGPQEPKDTSEQPRPSKAEPRPSTTPSTSLGPQEPKDTSEQPRPSKAEPRPSTTPST 300
Rom4.1_T2B      ------------------------------------------------------------ 211

Rom4.1_R1A      SLGPQEPKDTSEQPRPSKAEPRPSTTPSTSLGPQEPKDTSEQPRPSKAEPRPSTTPSTSL 360
Rom4.1_T2B      --------------------------------------------------------STSL 215
                                                                        ****

Rom4.1_R1A      GPQEPKDTSEQPRPSKAEPRPSTTPSTSLGPQEPKDTSEQPRSSKAESKLSTTLSKLRFS 420
Rom4.1_T2B      GPQEPKDTSEQPRPSKAEPRPSTTPSTSLGPQEPKDTSEQPRSSKAESKLSTTLSKLRFS 275
                ************************************************************

Rom4.1_R1A      RESRSVIKGTGSFGGSSQVSSNAGRRVHTLADTASTDDASIRQAMSQSFSSPGVSQPEQP 480
Rom4.1_T2B      RESRSVIKGTGSFGGSSQVSSNAGRRVHTLADTASTDDASIRQAMSQSFSSPGVSQPEQP 335
                ************************************************************

Rom4.1_R1A      ERASRGRNMFGMRRRPCWIRIQVKEKIFPGKM         512
Rom4.1_T2B      ERASRGRNMFGMRRRPCWIRIQVKEKIFPGKM         367
                ********************************
```

Supplementary Figure S6. Analysis of polymorphism and synonymous and non-synonymous substitutions in the repeat region of ROM4.5. **A**. Nucleotide sequence variants encoding for 29 aa-repeats in different *Babesia bovis* geographical isolates. The sequence shown for T2B is 100% conserved in all analyzed American isolates. When different sequences were found in an isolate, they are designated with letters. The results of the SNAP analysis to find the average dN/dS are shown below. **B**. Clustal omega alignment of the 29 aa peptides encoded by the nucleotide sequences shown in A. Asterisks at the bottom mark conserved positions between all sequences, colons mark conserved substitutions and absence of a sign corresponds to non-conserved substitutions.

A

T2B	GAACCTAAAGACACCAGCGAACAACCTAGGCCATCGAAAGCTGAACCTCGACCTAGTACCACACCATCTACCTCCTTAGGTCCACAA
SA2	GAACCTAAAGACACCAGCGAACAACCTAGGCCATCGAAAGCTGAACCTCGACCTAGTACCACACCATCTACTTCGTTAGGTCCACAA
I3A	GAAATGAAAGGTAGCAGTGAACAACCTAAGCCATCTAAAACTGAGTCTCGACCTAGTACTACACCGTCTACTTCGTTAGGTCCACAA
I3B	GAAGTTAAAGAAACTAGTGAACAACCTAAGCCATCTAAAACTGAGTCTCGACCTAGTACTACACCGTCTACTTCGTTAGGTCCACAA
I3C	GAAGTTAAAGAAACTAGTGAACAACCTAGGCCATCTAAAACTGAGTCTCGACCTAGTACTACACCGTCTACTTCGTTAGGTCCACAA
I3D	GAAGTTAAAGAAACTAGTGAACAACCTAGGCCATCTAAAACTGAGTCTCGACCTAGTACTGCACCGTCTACTTCGTTAGGTCCACAA
A1A	GAAATGAAAGGTAGCAGTGAGCAGCCTAGGCCATCGAAAGGTGAACCTCGTCCTAGTACCACACCATCTACTTCCTTAGGTCAACAA
A1B	GAAGCTAAAGACATTAGTGAGCAGCCTAGGCCATCGAAAGCTGAACCTCGTCCTAGTACCACACCATCTACTTCCTTAGGTCAACAA
A1C	GAAGCTAAAGACATCAGTGAAAAGCCTAGGCCATCGAAAGGTGAACCTCGTCCTAGTACCACACCATCTACTTCCTTAGGTCAACAA
A15A	GAAATGAAAGGTAGCAGTGAGCAGCCTAGGCCATCGAAAGGTGAACCTCGTCCTAGTACCACACCATCTACTTCCTTAGGTCAACAA
A15B	GAAGCTAAAGACATTAGTGAGCAGCCTAGGCCATCGAAAGCTGAACCTCGTCCTAGTACCACACCATCTACTTCCTTAGGTCAACAA
A15C	GAAGCTAAAGACATTAGTGAAAAGCCTAGGCCATCGAAAGGTGAACCTCGTCCTAGTACCACACCATCTACTTCCTTAGGTCAACAA
A15D	GAAGCTAAAGACATTAGTGAGCAACCTAGGCCATCGAAAGCTGAACCTCGACCTAGTACCACACCATCTACTTCCTTAGGTCCACAA

SNAP results: Averages of all pairwise comparisons: dS = 0.3854, dN = 0.0947, dN/dS = 0.24

B

```
A1C       EAKDISEKPRPSKGEPRPSTTPSTSLGQQ    29
A15C      EAKDISEKPRPSKGEPRPSTTPSTSLGQQ    29
T2B       EPKDTSEQPRPSKAEPRPSTTPSTSLGPQ    29
SA2       EPKDTSEQPRPSKAEPRPSTTPSTSLGPQ    29
A1B       EAKDISEQPRPSKAEPRPSTTPSTSLGQQ    29
A15B      EAKDISEQPRPSKAEPRPSTTPSTSLGQQ    29
A15D      EAKDISEQPRPSKAEPRPSTTPSTSLGPQ    29
A1A       EMKGSSEQPRPSKGEPRPSTTPSTSLGQQ    29
A15A      EMKGSSEQPRPSKGEPRPSTTPSTSLGQQ    29
I3A       EMKGSSEQPKPSKTESRPSTTPSTSLGPQ    29
I3D       EVKETSEQPRPSKTESRPSTAPSTSLGPQ    29
I3B       EVKETSEQPKPSKTESRPSTTPSTSLGPQ    29
I3C       EVKETSEQPRPSKTESRPSTTPSTSLGPQ    29
          * *  **:*:*** * ****:****** *
```

Supplementary Figure S7. Clustal omega alignment between N-terminal regions of ROM4.5 of the Mexican Mo7 strain and the Australian A15 strain of *Babesia bovis*. The repeat region is marked by square brackets. The conserved aa motifs flanking the repeats' region are in red.

```
ROM4.5_A15      MGDNDSNEKKNAGRHSLSAIKGGFVKDMVNRGFRKKVNDSDKNDEGKEDKSGDKPTNVSK 60
ROM4.5_Mo7      MGDNDSNEKKNAGRHSLNAIKGGFVKDMVNRGFRKKVNDSDKNDESKEDKGGDKPTNVSK 60
                *****************.***************************.****.*********

ROM4.5_A15      DTSSMKAGNVAALRDQFKRAVEKDESKDQNSQAGQSSLTSKAPLGDPRTDQRARAKFLAE 120
ROM4.5_Mo7      DTSSMKSGNVAALRDQFKRAVEKDDSKDQNSQAGQSSSTSKAPLGDPRTDQRARAKFLAE 120
                ******:*****************:************ **********************

ROM4.5_A15      KYEKILAARNMVEESASRALQRPATNTPAKIPPSVSGVSKEMKGSSEQPRPSKAEPRPST 180
ROM4.5_Mo7      KYEKILAARNMVEESASRALQRPATSTPAKIPPSVSGVSKEPKDTSEQPRPSKAEPRPST 180
                *************************.*************** *.:***************

ROM4.5_A15      TPSTSLGQQEAKDISEQPRPSKAEPRPSTTPSTSLGQQEAKDISEKPRPSKAEPRPSTTP 240
ROM4.5_Mo7      TPSTSLGPQE-------------------------------------------------- 190
                ******* **

ROM4.5_A15      STSLGQQEAKDISEQPRPSKAEPRPSTTPSTSLGPQEAKDISEQPRPSKAEPRPSTTPST 300
ROM4.5_Mo7      --------PKDTSEQPRPSKAEPRPSTTPSTSLGPQEPKDTSEQPRPSKAEPRPSTTPST 242
                         ** ************************* ** *******************

ROM4.5_A15      SLGPQEAKDISEQPRPSKAESKLSTALSKLRSSRESRSVIKGTGSFGGSSQVSSSAGRRV 360
ROM4.5_Mo7      SLGPQEPKDTSEQPRPSKAESKLSTTLSKLRFSRESRSVIKGTGSFGGSSQVSSNAGRRV 302
                ****** ** ***************:***** **********************.*****

ROM4.5_A15      HTLADTASTDDASIRQALSQSFSSPGVSQSEQPERGSRGRNMFGMRRRPCWIRIQVKEKI 420
ROM4.5_Mo7      HTLADTASTDDASIRQAMSQSFSSPDVSHPEQPERASRGRNMFGMRRRPCWIRIQVKEKI 362
                *****************:*******.**: *****.************************

ROM4.5_A15      FPGKM        425
ROM4.5_Mo7      FPGKM        367
                *****
```

Supplementary Figure S8. PCR and RT-PCR amplification of *Babesia bovis rom4.3/4.4*. DNA (g) or RNA (+ , -) extracted from AS (A) or SS (B) were used as templates for PCR and RT-PCR amplification of *rom4.3/4.4* genes or transcripts, respectively. M: DNA ladder; the arrows mark the 500 bp band; b: blank without DNA template; + reaction with reverse transcriptase; - reaction without reverse transcriptase. A band corresponding to the expected size (259 bp) was only obtained when reverse transcriptase was added to the RT-PCR reaction, showing that the AS and SS RNA preparations were free of DNA contamination.

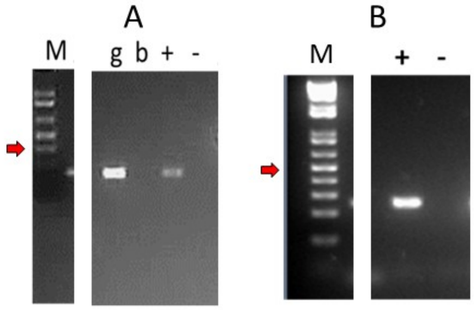

Supplementary Figure S9. Scheme of PCR amplification of r*om4* alleles for polymorphism analysis. A: *rom4.1*, B: *rom4.2*, C: *rom4.3/4.4*, D: *rom4.5*

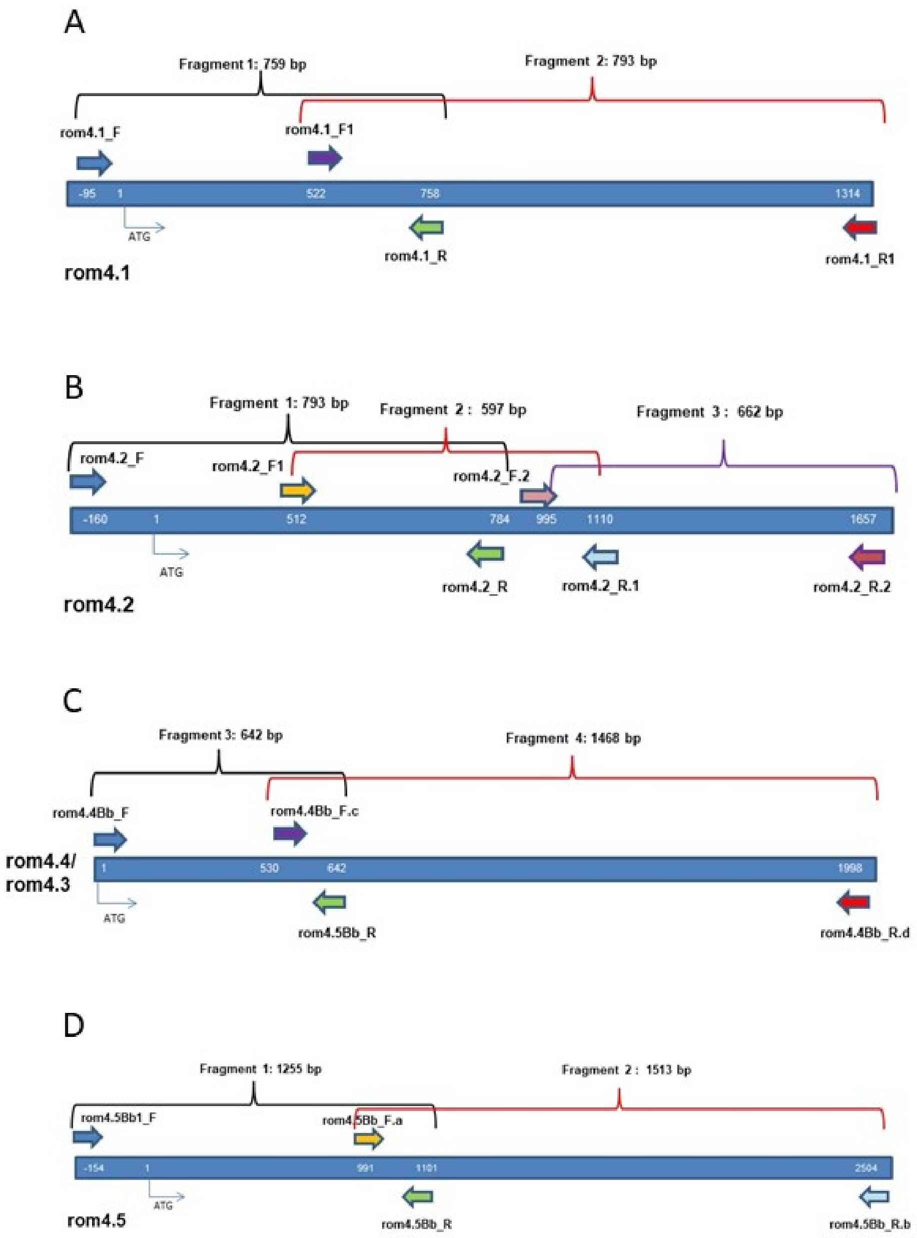

Supplementary Table S1. Oligonucleotides used in qRT-PCR

Gene	Primer name	Sequence	T (°C)	Length (bp)
rom4.1<br>BBOV_II006100	rom4.1_F<br>rom4.1_R	CACTCATATCGTGGGCATTG<br>CATGTTTTTGGGCCATATCC	59.9<br>59.8	125
rom4.2<br>BBOV_II006070	rom4.2_F<br>rom4.2_R	CAGGCGTACATTTTGGGTTT<br>AGGCGATCCCATCCTATACC	59.9<br>59.7	74
rom4.4<br>BBOV_II005940/<br>*rom4.3*<br>BBOV_II005950	rom4.2/3_F<br>rom4.2/3_R	TCGCATTCGTTAGCATTGAG<br>CGGTCAAACGCAGATACTGA	59.9<br>59.8	126
rom4.5<br>BBOV_II005930	rom4.5_F<br>rom4.5_R	GGCAACTTCACTTGCTCCTC<br>TAGTGCGCCAACAATAGTGC	60<br>59.9	64
topoisomerase II<br>BBOV_III004820	topo1_F<br>topo1_R	GGGATAGGTACAGGATTCAGC<br>TCCGTTTTCATCTAGCCACTG	61<br>64	197
	topo2_F<br>topo2_R	CAGGACAGAGGAAGGTGCTC<br>CCATGGTGGTATGCAGAGTG	60<br>60	109
actin<br>BBOV_IV009790	actin_F<br>actin_R	ACCAGGCTGAACCGCTACTA<br>TCATCAAAACGGAAGCTCCT	60<br>60	240
gadph<br>BBOV_II002540	gadph_F<br>gadph_R	TTGGTTCTCCGTGCTTCTTT<br>GGAATCTGAGAAGGCTCACG	60<br>60	209

Supplementary Table S2. Ct and efficiency values obtained by qPCR targeting potential reference genes of *Babesia bovis*. The housekeeping genes *topoisomerase*, *gadph* and *actin* were tested by qPCR using specific primers and (A) plasmid DNA and (B) AS or SS cDNA as template. The primers used are shown in Table S1. Each determination was done in triplicates (Ct1, Ct2 and Ct3). Efficiency was calculated using the absolute fluorescence increase method with LinRegPCR software.

A

Primer pair	Ct 1	Ct 2	Ct 3	Mean Ct	Mean efficiency	Mean efficiency %
topo 1	24.537	24.469	23.960	24.32	1.825	82.5
topo 2	18.009	18.367	18.153	18.18	1.881	88.1
gadph	22.199	21.832	22.207	22.08	1.807	80.7
actin	25.168	26.055	25.497	25.57	1.868	86.8

B

Primer pair	Mean CT of AS	Mean CT of SS	SS/AS ratio	Mean efficiency	Mean efficiency %
topo 1	32.99	30.72	0.93	1.936	93.6
topo 2	33.38	32.99	0.99	1.931	93.1
gadph	34.8	25.64	0.74	1.934	93.4
actin	31.65	35.52	1.12	1.934	93.4

Supplementary Table S3. Information on *Babesia bovis* strains and isolates used to study *rom4* sequence polymorphism

Isolate/strain name	Region and/or country of origin	Characteristics	Reference
T2Bo	Texas, USA	Pathogenic[a]	[51]
Mo7	Mexico	Pathogenic[a]	[52]
R1A	Salta, Argentina	Attenuated[a]	[53,54]
S2P	Salta, Argentina	Pathogenic[a]	[54,55]
M2P	Corrientes, Argentina	Mildly pathogenic	[54]
B4P	Rio Grande do Sul, Brazil	Pathogenic	[56]
I3	Gonen, Israel	Attenuated	[57]
A1 (F71 plus1)	North Queensland, Australia	Mildly pathogenic	[58]
A15 (H92)	Central Queensland, Australia	Pathogenic	[58]
SA2 (RLB18/061)	South Africa	unknown	-

Supplementary Table S4. Primers used to amplify *rom4* segments for polymorphism studies

Primer	Sequence	Length (bp)	Tm (°C)
rom4.1_F	CCTCCATAGTAGTTGGAAGC TC	579	58
rom4.1_R	ATGCGACGCGGAGTAACAC		61
rom4.1_F1	CTACGGCTAATCATCGTGTTC	793	57
rom4.1_R1	CGGGCATGTAGTTCGTAATC		56
rom4.2_F	GATTGTATTGCGAACGGCACTG	793	61
rom4.2_R	CTCAACACAGTACGGTGCAA 3		58
rom4.2_F1	GGGTTGTGGAAGCTATCTAGCTG	597	61
rom4.2_R1	GTGAATCGGTAGTATGGAACTCC		58
rom4.2_F2	TGGCATAACGACACAATATTGTT	662	57
rom4.2_R2	ACAACGAAAGAGAACCAGGA		57
rom4.4Bb_F	ATGGGAGATAATGATTCGAATG	642	62
rom4.5Bb_R	CATCTTACCGGGAAATATCTTC		54
rom4.4Bb_F.c	TCAGCCAGAGCAACCCGAG	1468	62
rom4.4Bb_R.d	TTATTTCTCACAAAAAACATTCCA		54
rom4.5Bb1_F	TGCGAGATATGCAACTCACGA	1255	60
rom4.5Bb_R	CATCTTACCGGGAAATATCTTC		54
rom4.5Bb_F.a	AGCCAGAGCAACCCGAGCG	1513	68
rom4.5Bb_R.b	AATGATGAAAAGTTCAGCATGTA		55
